# The Genomic Architecture of Population Divergence between Subspecies of the European Rabbit

**DOI:** 10.1371/journal.pgen.1003519

**Published:** 2014-08-28

**Authors:** Miguel Carneiro, Frank W. Albert, Sandra Afonso, Ricardo J. Pereira, Hernan Burbano, Rita Campos, José Melo-Ferreira, Jose A. Blanco-Aguiar, Rafael Villafuerte, Michael W. Nachman, Jeffrey M. Good, Nuno Ferrand

**Affiliations:** 1 CIBIO, Centro de Investigação em Biodiversidade e Recursos Genéticos, Campus Agrário de Vairão, Vairão, Portugal; 2 Departamento de Biologia, Faculdade de Ciências Universidade do Porto, Porto, Portugal; 3 Department of Evolutionary Genetics, Max Planck Institute for Evolutionary Anthropology, Leipzig, Germany; 4 Princeton University, Lewis Sigler Institute for Integrative Genomics, Princeton, New Jersey, United States of America; 5 Marine Biology Research Division, Scripps Institution of Oceanography, University of California San Diego, La Jolla, California, United States of America; 6 Department of Molecular Biology, Max Planck Institute for Developmental Biology, Tuebingen, Germany; 7 IREC, Instituto de Investigación en Recursos Cinegéticos (CSIC-UCLM-JCCM), Ciudad Real, Spain; 8 Instituto de Investigación en Recursos Cinegéticos (CSIC-UCLM-JCCM), Departamento de Zoología, Universidad de Córdoba, Córdoba, Spain; 9 Department of Ecology and Evolutionary Biology, University of Arizona, Tucson, Arizona, United States of America; 10 Division of Biological Sciences, The University of Montana, Missoula, Montana, United States of America; University of Notre Dame, United States of America

## Abstract

The analysis of introgression of genomic regions between divergent populations provides an excellent opportunity to determine the genetic basis of reproductive isolation during the early stages of speciation. However, hybridization and subsequent gene flow must be relatively common in order to localize individual loci that resist introgression. In this study, we used next-generation sequencing to study genome-wide patterns of genetic differentiation between two hybridizing subspecies of rabbits (*Oryctolagus cuniculus algirus* and *O. c. cuniculus*) that are known to undergo high rates of gene exchange. Our primary objective was to identify specific genes or genomic regions that have resisted introgression and are likely to confer reproductive barriers in natural conditions. On the basis of 326,000 polymorphisms, we found low to moderate overall levels of differentiation between subspecies, and fewer than 200 genomic regions dispersed throughout the genome showing high differentiation consistent with a signature of reduced gene flow. Most differentiated regions were smaller than 200 Kb and contained very few genes. Remarkably, 30 regions were each found to contain a single gene, facilitating the identification of candidate genes underlying reproductive isolation. This gene-level resolution yielded several insights into the genetic basis and architecture of reproductive isolation in rabbits. Regions of high differentiation were enriched on the X-chromosome and near centromeres. Genes lying within differentiated regions were often associated with transcription and epigenetic activities, including chromatin organization, regulation of transcription, and DNA binding. Overall, our results from a naturally hybridizing system share important commonalities with hybrid incompatibility genes identified using laboratory crosses in mice and flies, highlighting general mechanisms underlying the maintenance of reproductive barriers.

## Author Summary

A number of laboratory studies of speciation have uncovered individual genes that confer lower fitness when in a divergent genetic background. It is, however, unclear whether these genes were ever relevant to restricting gene flow in nature, or whether they are indirect consequences of functional divergence between species that in most cases can no longer hybridize in natural conditions. Analysis of introgression across the genome of divergent populations provides an alternative approach to determine the genetic basis of reproductive isolation. Using two subspecies of rabbits as a model for the early stages of speciation, we provide a genome-wide map of genetic differentiation. Our study revealed important aspects of the genetic architecture of differentiation in the early stages of divergence and allowed the identification of genomic regions that resist introgression and are likely to confer reproductive barriers in natural conditions. The gene content of these regions, which in several cases reached gene-level resolution, suggests an important role for epigenetic and transcription regulation in the maintenance of reproductive barriers. Some of the genes identified here in a natural system are similar in function to the hybrid male sterility genes identified in laboratory studies of speciation.

## Introduction

Most research into the genetic underpinnings of speciation is based on laboratory studies of specific reproductively isolating phenotypes [1], [2]. A small number of these studies have identified individual genes that confer lower fitness when in a foreign or admixed genetic background [3]–[6]. However, it is not clear whether these genes and corresponding phenotypes - often identified in crosses between highly divergent species pairs - were ever relevant to restricting gene flow in nature, or whether they simply represent an inevitable byproduct of functional divergence between species [1], [7]. Moreover, it is not clear that hybrid incompatibilities accumulated long after the cessation of gene flow are strongly representative of the early stages of speciation. The direct analysis of naturally hybridizing populations provides an alternative and arguably more direct way to study the genetic basis of reproductive isolation [8]. This approach assures relevance to the early stages of speciation and allows the fitness of hybrid genotypes to be evaluated under natural conditions [9], [10].

Natural hybridization has been extensively studied in the last few years revealing the mosaic nature of gene flow across species boundaries [11]. It is now well established that genomic regions harboring hybrid incompatibilities are expected to display low levels of introgression, whereas regions not harboring incompatibilities should introgress more freely [12]–[14]. In spite of these general expectations, the identification of individual genes causing reproductive isolation in nature has remained elusive for at least three reasons. First, many hybrid zone studies have lacked mapping resolution due to limited sampling of the genome. Second, numerous historical and biological factors can inherently limit the fine-scale resolution of individual incompatibilities within many hybrid zones. For example, time since secondary contact, rates of dispersal and hybridization, and genomic architecture will all influence the effective rate of recombination and thus mapping resolution within a hybrid zone. Third, in many species, widespread differentiation across much of the genome limits the ability to detect loci contributing to isolation. In principle, some of these limitations can be overcome by applying high-throughput sequencing to the study of introgression between partially-isolated populations that are characterized by a long history of extensive gene flow. Genome-wide data from such populations promises to provide insight into the genetic basis of reproductive isolation in nature [15]–[19] and thereby provide a powerful counterpart to laboratory studies of speciation.

Two naturally hybridizing subspecies of the European rabbit (*Oryctolagus cuniculus algirus* and *O. c. cuniculus*) are a good model for the early stages of reproductive isolation. These two subspecies currently occur in parapatry, hybridize over a large area in the central part of the Iberian Peninsula (Figure S1), and have diverged approximately 1.8 million years ago [20], [21]. The current secondary contact is thought to result from a range expansion associated with the amelioration of climatic conditions after the last glacial maximum (∼18000 ya) [20], although previous glacial/interglacial cycles during the Pleistocene may have provided multiple opportunities for vicariant and past hybridization events. Both *O. c. algirus* and *O. c. cuniculus* span the three major climatic zones in the Iberian Peninsula (Mediterranean, Oceanic, and semi-arid) with little evidence for morphological differentiation along these ecological transitions [22], . The two subspecies can hybridize in the lab and produce viable hybrids, but hybrid males suffer from reduced fertility (Ferrand, unpublished results). Genetic studies using samples along the distribution range of both subspecies and based on a few dozen nuclear markers have revealed loci of relatively high divergence (0.3–1.2%) between subspecies embedded in a genome otherwise characterized by low levels of differentiation and high levels of bidirectional gene flow [21], [24]–[26], likely facilitated by high effective population sizes, high dispersal, and a relatively short generation time [21], [24]–[26]. Differentiation appears most pronounced on the sex chromosomes and in centromeric regions, albeit based on limited and biased genomic sampling.

A more recent study using individuals sampled across the hybrid zone and 22 markers showed that some loci are characterized by concordant and stepped clines that do not co-localize with any obvious physical or ecological barriers [27], suggesting an overall barrier to gene flow likely to be primarily maintained by selection against intrinsically unfit hybrids. This same study revealed large inter-locus variation in cline width and cline center among some loci, consistent with substantial variation in patterns and levels of introgression as indicated by previous work [21], [24]–[26]. Long tails of introgression were often detected such that populations from the extreme ends of the Iberian Peninsula, which are well removed from the primary hybrid zone, were not genetically pure [27] (Figure S2). Thus, introgression seems not only to affect a large portion of the genome but frequently occurs through the entire range of parental populations. These previous studies provided important information regarding the dynamics of contact and hybridization. However, they were not designed to delineate regions of the genome resisting introgression nor investigate the frequency and types of genes involved in genetic incompatibilities, because they were based on a relatively small number of loci (less than 50) and the sampling of the genome was non-random (e.g. overrepresentation of the X-chromosome or regions near centromeres).

The combination of extensive introgression between rabbit subspecies together with the availability of a rabbit reference genome sequence provides ideal conditions to resolve the genetic basis and architecture of persistent reproductive barriers between hybridizing taxa in the early stages of divergence. In this study, we used Illumina sequencing to provide a high-resolution map of genetic differentiation between rabbit subspecies. Our main goal was to identify specific genes or genomic regions that have resisted introgression and are likely to confer reproductive barriers in natural conditions. We further investigated the proportion of the genome that is resisting introgression and the number and size of such regions. We also tested whether previously hypothesized positional effects on genetic differentiation between rabbit subspecies (i.e. centromeres and X-chromosome) are robust to a more dense and unbiased sampling of the genome. We used custom microarrays to capture and Illumina sequence 6 Mb of targeted intronic DNA, and combined this dataset with published protein coding sequences derived from transcriptome sequencing [28]–[29]. The combination of genome-wide data and population genetic inference allowed the fine-scale identification of genes likely to underlie reproductive barriers in nature.

## Results/Discussion

### Low to moderate overall levels of genetic differentiation between subspecies

We analyzed a large dataset consisting of ∼326,000 SNPs (∼177,000 from the targeted DNA capture of intronic sequence and ∼149,000 from the transcriptome resequencing) distributed on all rabbit chromosomes, providing an unbiased picture of genetic differentiation between rabbit subspecies at the genome-wide level. This dataset was obtained in rabbits sampled at some distance from the primary contact zone (Figure S1), but in geographic regions that nonetheless show high levels of introgression [21], [24]–[27] (see Figure S2).

**Figure 1:**
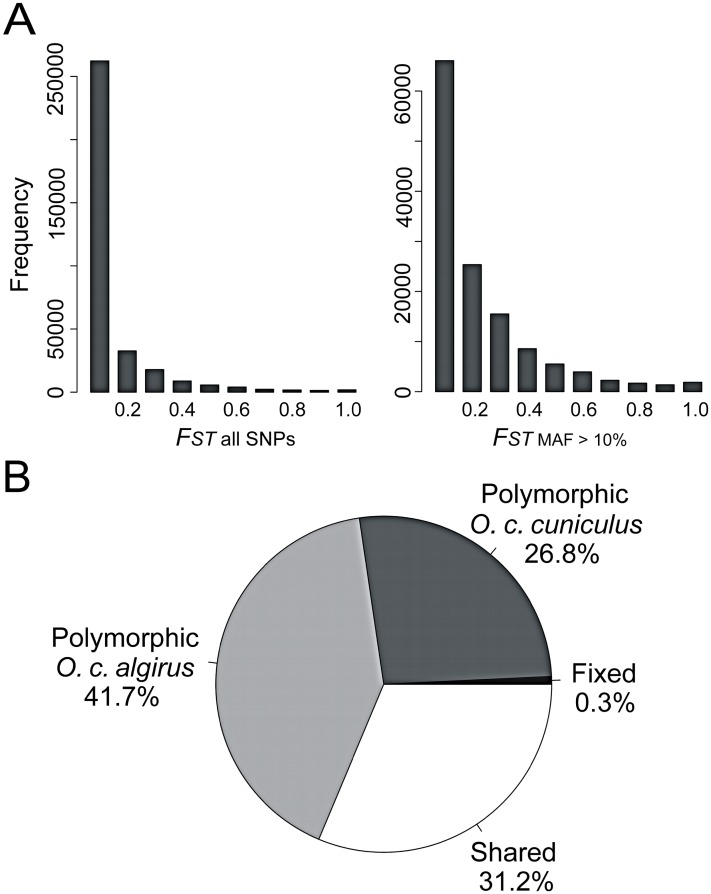
Low to moderate overall genetic differentiation between the two subspecies of rabbits. A) Histogram of *F_ST_* values for all SNPs and SNPs showing a minor allele frequency (MAF) of 10%. B) Pie chart summarizing the proportion of fixed, shared and exclusive polymorphisms.

We found that overall levels of genetic differentiation between the rabbit subspecies were low to moderate (Figure 1). Fixed differences in our dataset accounted for 0.3% of the total number of mutations, and shared polymorphisms were ∼100 fold more numerous (31.2%). The percentage of fixed differences in nature may be even lower given the relatively small number of individuals used in our study. We estimated an average fixation index (*F_ST_*) value of 0.084 across all polymorphisms and of 0.169 across polymorphisms with a minor allele frequency (MAF) threshold of 10% (132,199 SNPs). Thus, the overall baseline of differentiation is moderate even after excluding low frequency variants, which are more likely than high-frequency variants to represent sequencing and genotyping errors [30]. The mean levels of differentiation reported here (*F_ST_*_autosomes_ = 0.082; *F_ST_*_X-chromosome_ = 0.185) were lower than previously reported values using Sanger sequencing of small intronic fragments (*F_ST_*_autosomes_ = 0.146; *F_ST_*_X-chromosome_ = 0.447) [26] and remained lower for the X-chromosome after the removal of low frequency variants (*F_ST_*_autosomes MAF>10%_ = 0.164; *F_ST_*_X-chromosome MAF*>*10%_ = 0.368). This difference likely reflects the biased sampling of the genome in previous studies towards regions of higher differentiation (e.g. centromeric regions). For comparison, our genome-wide estimates in rabbits are similar to that between human population groups, namely Africans, Caucasians, and Asians (*F_ST_*≈0.120) [31].

There are very few examples in the literature describing such low levels of genetic differentiation in the context of hybridizing taxa showing partial reproductive barriers. *Anopheles* mosquitoes provide perhaps one of the most extreme examples. Recent results describe numerous regions of differentiation between forms but large portions of the genome remain lowly differentiated [16], [32], [33]. However, they are thought to have diverged very recently - within the past 10,000 years - associated with the development of agriculture. In contrast, genetic divergence at multiple loci suggests that rabbits started diverging approximately 1.8 million years ago [20],[21]. A more comparable example is found in sunflowers. A recent study found that genetic differentiation was much lower throughout a large fraction of the genome between the now sympatric species pair *Helianthus annuus* and *H. petiolaris*, which have diverged for ∼1.0 million years, than between more closely related species with non-overlapping geographical ranges [34], [35].

The shared variation between rabbit subspecies is likely due to both gene flow and retention of ancestral variation. Evidence for high levels of gene flow comes from several sources. First, previous estimates of gene flow between rabbit subspecies using an Isolation-with-Migration model [36], [37] and a similar sampling scheme to the one used here suggest moderate to high levels of gene exchange (2*Nm*≈1.2 for the autosomes averaged in both directions) [26]. These estimates are at the higher end of the range of values reported in a recent review of similar studies of hybridizing taxa [11]. Second, the spatial distribution of allelic frequencies across the hybrid zone revealed a subset of coincident, narrow and stepped clines consistent with reproductive barriers acting to prevent introgression in a fraction of the genome, but also that cline width varied by a factor of 50 with introgressed alleles often reaching the distribution ends of both subspecies [27] (Figure S2). Evidence for unsorted ancestral variation comes from a consideration of the divergence time and the effective population size (*N_e_*). Effective population sizes (*N_e_*) in rabbits are on the order of 10^6^ [21]. Since the average coalescence time for alleles within a population is 4*N_e_* generations for the autosomes and 3*N_e_* for the X-chromosome, ancestral variation is still expected to be segregating between subspecies, even in the absence of gene flow (*N_e_*≈10^6^, divergence time≈1.8 Mya, and a generation time of one year) [21], [38].

It is exceptionally difficult to resolve the relative contribution of these two forces in structuring genetic variation between the subspecies. Nonetheless, given high levels of gene flow (2*Nm*>1) and persistence of ancestral variation, fixed differences between the subspecies are expected to be very rare across the genome. To quantify this expectation given the number of individuals sampled, we conducted simulations to estimate the expected number of fixed differences between the subspecies assuming previous estimates of the degree of gene flow [21], . We found that both the autosomes and X-chromosome were significantly enriched for fixed differences relative to the simulated neutral expectation (*P*<0.0001; Figure S3). Thus, genome-wide patterns of variation are characterized by extremely low levels of differentiation punctuated by an unusually high number of fixed differences. Given this pattern, genomic regions that are enriched for fixed differences are likely to be maintained by selection against introgression and thus contain genes involved in hybrid incompatibilities (or loci experiencing selection at linked sites within subspecies; see below).

### Multiple, independent differentiated regions across the genome suggest a complex genetic basis underlying reproductive barriers

**Figure 2:**
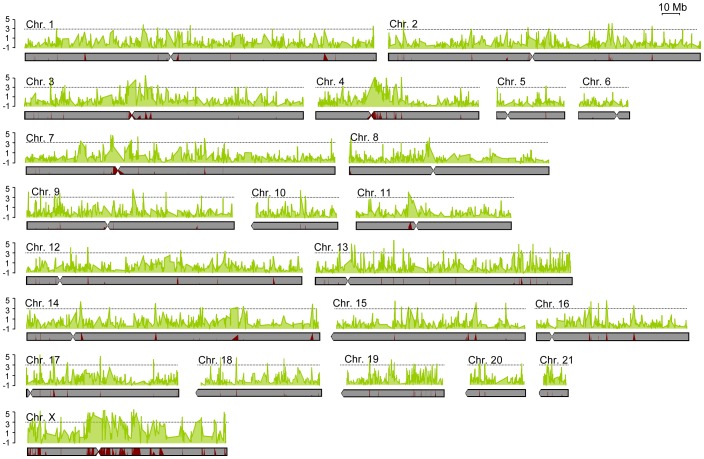
Levels of differentiation between subspecies varied greatly across the genome. Chromosomal distribution of genetic differentiation for all rabbit chromosomes. Each data point is based on a sliding window analysis using 100 kb windows with 10 kb steps. The solid black line denotes a Z-score of three (three standard deviations departing from the median *F_ST_* over all windows). The relative proportion of fixed (red) versus shared (gray) polymorphism for each window is indicated below the graph (F/S).

To investigate patterns of differentiation across the genome we first used a 100 kb sliding window approach (Figure 2; values are detailed in Dataset S1). Levels of differentiation varied greatly across the genome. Similar to our results for individual SNPs, the great majority of windows showed low to moderate differentiation (median *F_ST_*_ MAF>10%_ = 0.139), and extended portions of the genome were completely devoid of fixed differences. Several windows, however, were strongly differentiated relative to the genome-wide average. For the following analyses, we used SNPs with MAF>10%, although similar results are obtained when all SNPs are used (see Materials and Methods). For each window, we calculated the Z-score or the number of standard deviations departing from the median *F_ST_* over all windows, the proportion of fixed differences versus shared polymorphisms, and also the absolute number of fixed differences. 3.2% of windows showed a Z-score greater than three, which corresponded to an *F_ST_* value of 0.590 or higher. Intervals defined by a high Z-score also tended to show an enrichment of the absolute number of fixed differences and the proportion of fixed differences relative to shared polymorphisms (Figure 2). We found a 100% overlap between windows falling in the top 1% of the distribution of the absolute number of fixed differences and windows showing a significantly elevated proportion of fixed differences to shared polymorphisms when compared to the genome-wide average. We then used a randomized permutation test across SNPs within the genome (1000 replicates) to test if the clustering in levels of differentiation was unusual, given the genome-wide distribution of *F_ST_*_MAF>10%_ values for individual SNPs and fixed differences. None of the random permutations exceeded the observed differentiation (*P*<0.001), indicating that these regions of differentiation are unlikely to reflect simple stochastic variation in differentiation across the genome due to the reduced number of individuals sampled.

The spatial distribution of differentiation across the genome is potentially informative about the genetic architecture of reproductive isolation. To further demarcate contiguous genomic regions of high differentiation (i.e., genomic islands of differentiation), we identified all single or consecutive windows with significantly elevated ratios of fixed differences to shared polymorphisms. Using these criteria, we identified 140 independent regions of differentiation that together encompass ∼46.8 Mb of genomic sequence (∼1.8% of the genome; Table S1). Alternatively, we identified 173 regions (60.6 Mb and ∼2.3% of the genome; Table S2) when we used a Z-score of three or higher to define highly differentiated regions. Importantly, we found a high degree of overlap between these two metrics of differentiation (102 overlapping regions) (Table S3) and most differentiated regions were well demarcated (i.e. appearing as single peaks surrounded by background levels of much lower differentiation; Figure 2).

**Figure 3:**
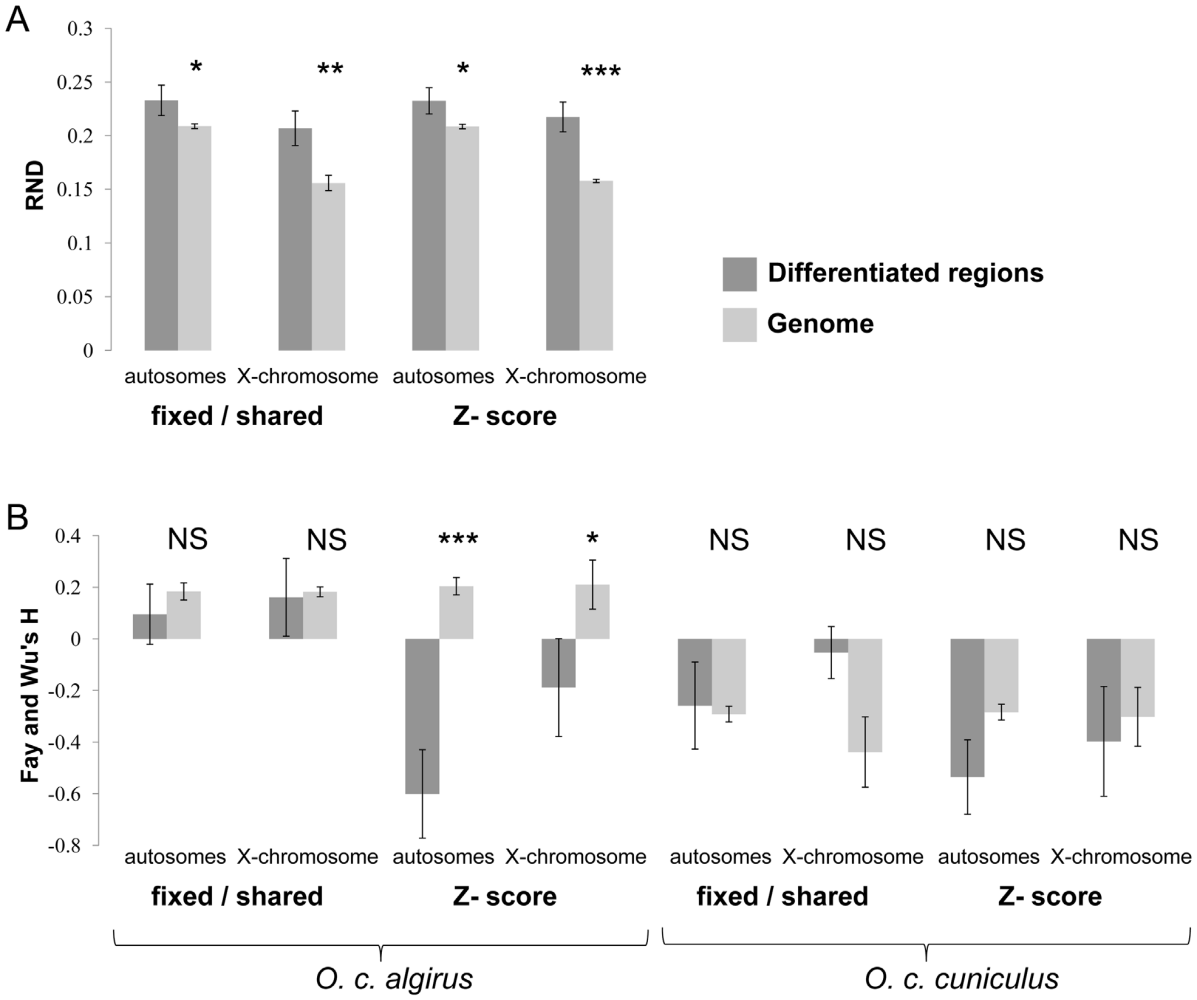
Regions of high differentiation are enriched for regions undergoing low rates of gene exchange. Mean RND (A) and Fays and Wu's *H* values (B) for intronic fragments contained within and outside regions of differentiation. Regions of differentiated were defined both based on the proportion of fixed differences to shared polymorphisms and on a Z-score higher than three (three standard deviations departing from the median *F_ST_* over all windows)). N.S. – non significant. Asterisks designate significant differences (t-test) for each comparison (*P<0.05; **P<0.01; ***P<0.001).

Our approach to identifying islands of divergence relies, in part, in levels of variation within populations and therefore is potentially sensitive to any process that reduces diversity within either subspecies, and thus high divergence may not be directly associated with signatures of reduced gene flow due to incompatibilities. For example, regions of high differentiation may be identified in our study due to selection at linked sites acting independently in each subspecies reducing within population variation relative to divergence between populations [39], [40]. We used two separate approaches to address this issue. First, we used a measure of differentiation, relative node depth (RND) [41], which does not depend on a within-population component of variation and therefore should be useful for distinguishing between both scenarios [42], [43]. RND takes into account possible mutation rate differences among genomic regions by incorporating divergence to a third more distantly related species, and is expected to be inversely proportional to the amount of gene flow. To generate appropriate outgroup data, we used a custom microarray to capture and sequence the same 5,000 introns used for rabbits in a single Hare (*Lepus timidus*) and then calculated RND for each region. We found much higher variance in RND values for intronic fragments consisting of a reduced number of sites, likely due to stochastic variation in the mutation process. To reduce sampling noise, we restricted our analyses to the 1,665 fragments with more than 1.0 kb of aligned *Lepus* data. Consistent with variable levels of introgression playing a substantial role in determining patterns of genomic differentiation, we found a significant increase in mean RND values for fragments residing within regions of differentiation defined both using the Z-score or the proportion of fixed differences versus shared polymorphisms (*P*<0.05 for all comparisons; Figure 3).

Second, we tested for evidence of more frequent selection within islands of differentiation. If regions of high differentiation reflect the independent action of positive selection within either subspecies (i.e., subspecific selective sweeps), then we would expect an excess of high frequency derived alleles within regions of differentiation [44]. We did not find significant differences or deviations in a consistent direction in the frequency of derived alleles in islands of divergence within *O. c. cuniculus* or in either subspecies when defining differentiation based on the proportion of fixed differences versus shared polymorphisms (Figure 3); however, intervals of high *F_ST_* in *O. c. algirus* did show a significant skew towards high frequency derived alleles. Our results do not rule out that a simple hitchhiking model or a combination of both factors (i.e. hitchhiking plus reduced introgression) may explain some of the regions of differentiation, particular within *O. c. algirus*. Indeed, several admixture scenarios [44], [45] and selection at linked sites are expected to generate an excess of high frequency derived alleles, but the observation that most islands of differentiation are not enriched for high frequency derived alleles suggests that selection at linked sites is likely to have a relatively small contribution overall to the occurrence of areas of high differentiation. Moreover, the fact that the inferred skew towards high frequency derived alleles was only detected in one subspecies is mostly consistent with asymmetric migration. Taken together, our results indicate that both *F_ST_* (i.e. Z-score) and the ratio of shared polymorphisms to fixed differences are capturing signatures of reduced gene flow and that regions of high differentiation indentified in our study are likely to be enriched for regions involved in reproductive barriers. Given that combining different summary statistics is likely to reduce the number of false positives, for the remainder of the analysis we defined highly differentiated regions as the 102 genomic intervals that show significant differentiation for both metrics.

Our results further show that signatures of reduced introgression are spread across the genome, suggesting that reproductive barriers in rabbits appear to have a complex genetic basis controlled by many unlinked genes. Genome-wide patterns of isolation do not provide direct information regarding the mechanisms or phenotypes that contribute to reproductive barriers, underscoring both the power of this population genetic approach because it is not constrained by choice of a specific phenotype, but also its limitations. Information regarding hybrid phenotypes in rabbits is scarce because wild animals are exceptionally difficult to maintain in captivity. Nonetheless preliminary data from crosses between subspecies indicate that hybrid males are less fertile than hybrid females or parental individuals. Hybrid male sterility often evolves very quickly and may show a complex genetic basis even at the earliest stages of divergence [e.g. 46], which could account for reduced introgression at multiple regions of the genome as inferred here. Yet, we cannot discard that other hybrid phenotypes, both resulting from intrinsic or extrinsic causes, also act as reproductive barriers between rabbit subspecies in nature.

### Islands of differentiation were highly variable in size and overrepresented on the X-chromosome and near centromeres

**Figure 4:**
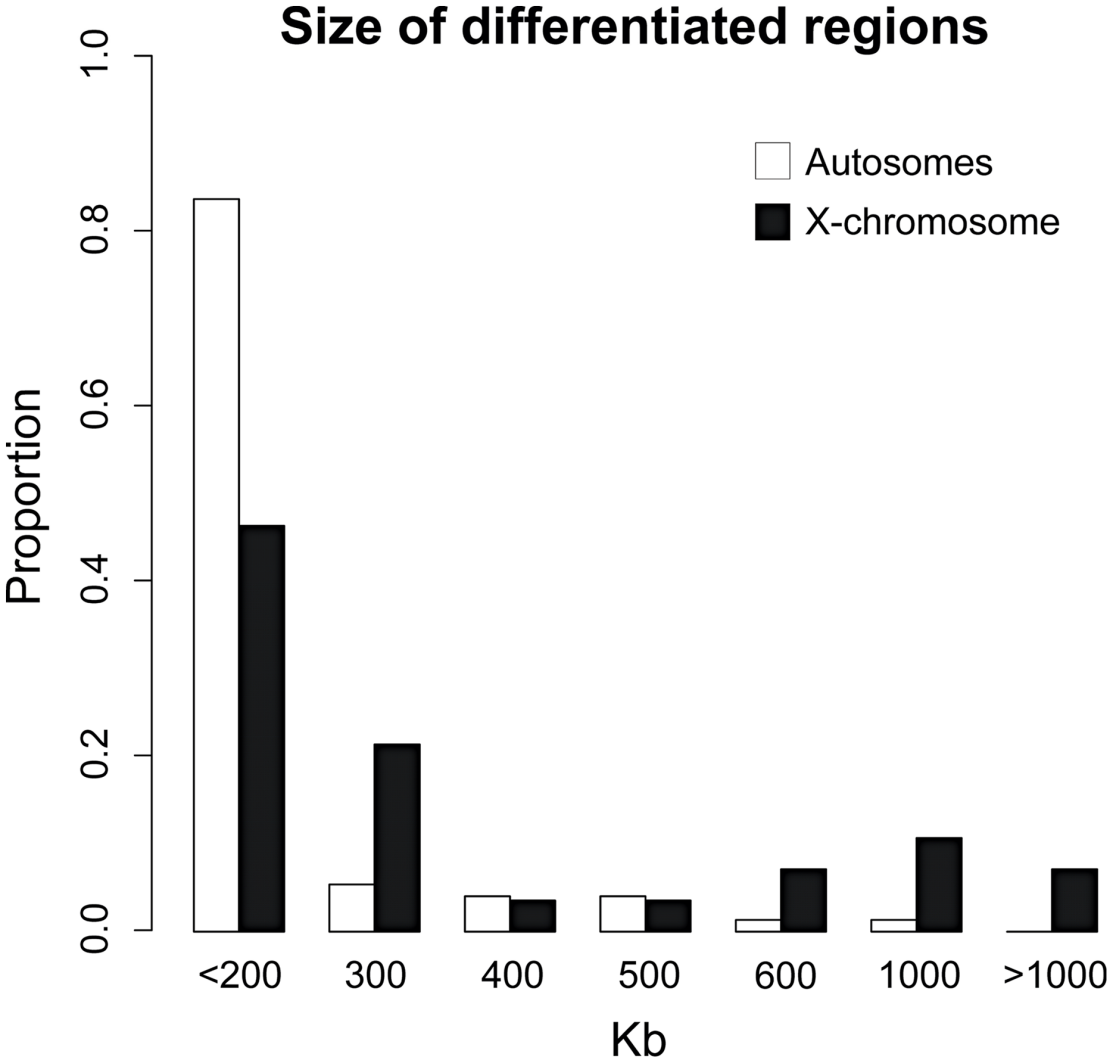
Highly heterogeneous size of differentiated regions. Histogram summarizing the physical size (Kb) of independent regions of differentiation between rabbit subspecies.

The physical size of differentiated regions was highly heterogeneous (Figure 4). The largest region of differentiation spanned more than ∼1.3 Mb but the majority of differentiation occurred on a physical scale smaller than 200 kb. The large number of small and independent regions of elevated differentiation in a background of low differentiation indicates that the time since secondary contact and subsequent hybridization has been sufficient for recombination to decouple most of the genome from adjacent isolation factors. Since the average gene density in a mammalian genome is ∼1 gene per 100 Kb, this scale of differentiation approaches gene-level resolution (see below).

Differentiated regions and their size were not randomly distributed across the genome. First, the X-chromosome exhibited particularly strong genetic differentiation (Figure 2). Mean *F_ST_* values were significantly higher on the X-chromosome (*F_ST_*_MAF>10%_ =  0.368) when compared to the autosomes (*F_ST_*_MAF>10%_ =  0.164; Mann-Whitney U test, *P*<0.001), as was the ratio of fixed to shared polymorphisms (0.283 vs 0.007; Fisher's Exact Test, *P*<0.001). This elevated differentiation was reflected in a significantly larger size of differentiated regions on the X than that on the autosomes (Mean_Autosomes_ = 202 Kb; Mean_X-chromosome_ = 379 Kb; Mann-Whitney U test *P*<0.001; Figure 4). Moreover, while the X-chromosome represents ∼5% of the rabbit genome, it contained a significant enrichment of differentiated regions (27.5%, *P*<0.001). In contrast, the mean RND value was significantly lower for the X-chromosome (RND = 0.176) when compared to the autosomes (RND = 0.209; *P*<0.001). A similar pattern between chimpanzees and humans has been interpreted as evidence for complex speciation scenarios involving differential gene flow [47], but several authors have pointed out that several other neutral and simpler explanations were not considered [e.g. 48]. RND values are corrected for mutation rate differences among loci using divergence to an outgroup; however, the X-chromosomes spends only one third of its evolutionary history in males. Thus, variation in the strength of male-biased mutation across time due to changes in reproductive system or generation time in the rabbits versus *Lepus* comparison could contribute in part to the autosome/X-chromosome difference. Additionally, the ancestral *N_e_* is expected to be smaller for the X chromosome than for the autosomes and this is expected to lead to lower RND for the former than for the latter.

**Figure 5:**
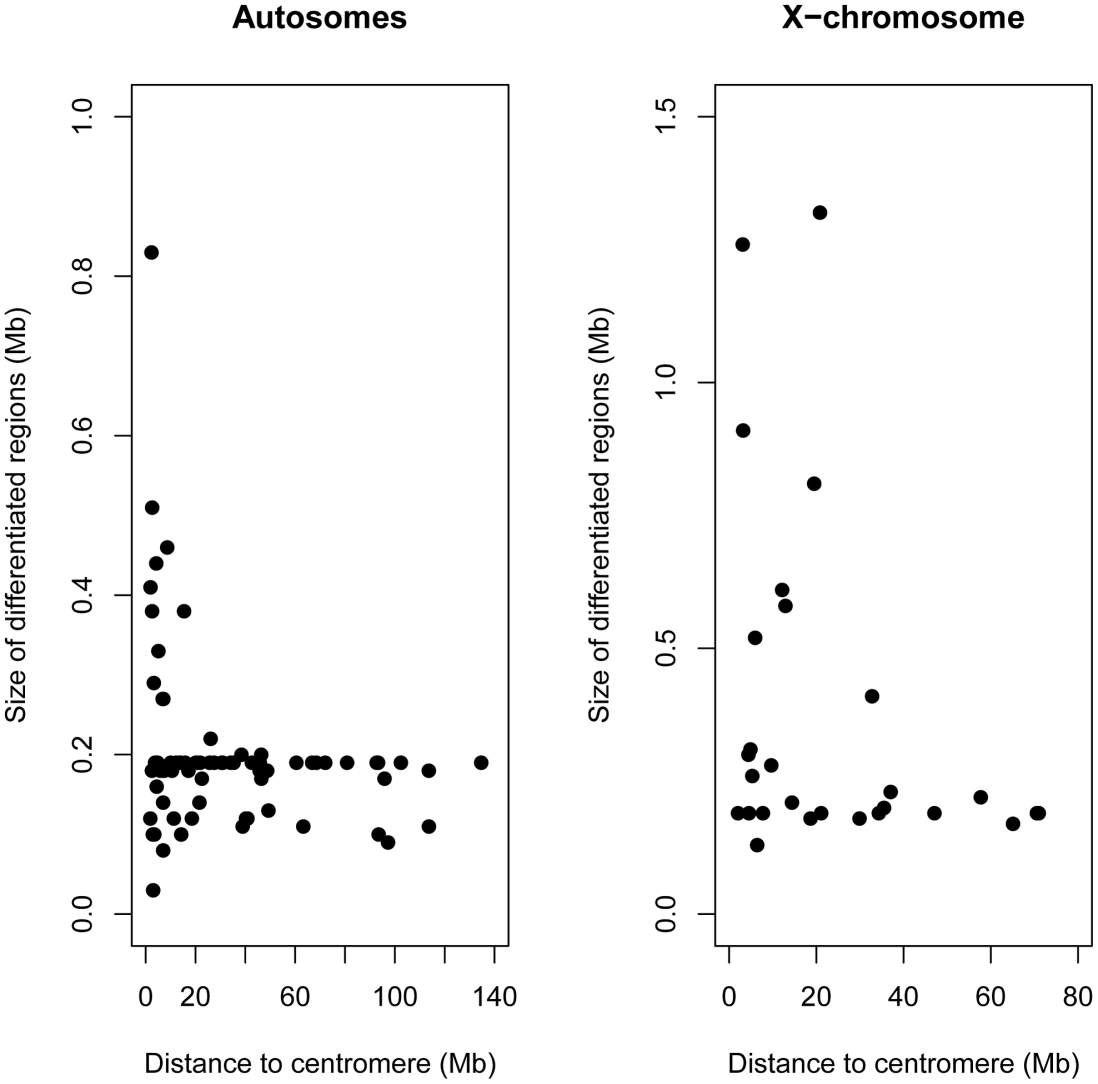
Larger regions of differentiation were found close to centromeres. Size of differentiated regions (Mb) relative to distance to the centromere (Mb) both for the autosomes (left panel) and X-chromosome (right panel).

Second, we observed strong differentiation close to several centromeric regions, which are known to have reduced rates of recombination in several species [e.g. 49]. Mean *F_ST_* and RND were significantly higher within 5 Mb of centromeres (*F_ST_*_MAF>10%_ =  0.246; RND = 0.228) when compared to the remainder of the genome (*F_ST_*_MAF>10%_ =  0.164; RND = 0.213; Mann-Whitney U test, *P*<0.05 for both tests). Two of the most notable such regions are found on chromosomes 4 and X (Figure 2). Regions of differentiation found within 5 Mb of centromeres were larger when compared to the rest of the genome (Mean_centromeres_ = 339 kb; Mean_genome_ = 225 kb; Mann-Whitney U test *P*<0.07; Figure 5), and significantly more numerous since they represent ∼9% of the genome and contain 22.5% of the total number of differentiated regions (*P*<0.01). We note that differentiation was not universally elevated near centromeres and many differentiated regions were found along chromosomal arms (Figure 2), including some larger than 500 Kb (Figure 5).

These genome-wide data confirm and extend previously described positional effects on genetic differentiation in rabbits [21], [24]–[26]. These findings underscore the central role that the X chromosome plays in the evolution of reproductive isolation [2], [50], and support the theoretical prediction that low recombination might facilitate species divergence in the face of gene flow [reviewed in 51]. According to these hypotheses, these regions should be enriched for genes affecting reproductive isolation. However, we cannot exclude the possibility that some of the regions of higher differentiation on the X or in centromeric regions are driven by selection at linked sites or reduced *N_e_* [39], [40].

### Insights into the genetic basis of speciation in nature

The availability of an annotated rabbit genome provides a good opportunity to access candidate genes within differentiated regions. Overall, these regions contained 410 known or predicted genes (Table S3), and of these genes, 337 were annotated as protein-coding genes, 2 as retrotransposed genes, 57 as noncoding RNAs, and 14 as pseudogenes. Consistent with the variation in size we also found a heterogeneous number of genes contained within each region (ranging from 1–31). Nine regions (8.8%) contained more than 10 genes each. In such regions it will be difficult to identify candidate variants underlying reproductive isolation. However, most differentiated regions contained very few annotated genes: 59 segments (57.8%) contained three or fewer genes and 30 (29.4%) localized to a single gene. Thus, while many regions throughout the genome appear to contribute to reproductive isolation, the substrate of selection within most regions is likely to be one or a few genes. These findings further underscore the utility of the rabbit hybrid zone for fine-scale mapping of candidate genes likely to be involved in the early stages of reproductive isolation.

Due to the reduced number of genes within most islands of isolation, our results allow us to explore the functional underpinnings of putative reproductive barriers at a higher resolution than most comparable genomic studies. We found that genes within islands of differentiation were enriched for several functional classes (Table S4), and most terms were related to transcription and epigenetic pathways (e.g. chromatin organization and modification, regulation of transcription and translation, DNA binding). These results are consistent with the emerging paradigm that epigenetic and transcription regulation frequently underlies the evolution of postzygotic hybrid dysfunctions [4], [5], [52], [53]. Contrary to several case studies on hybrid incompatibility genes [54]–[57], we found no statistical evidence for more rapid protein evolution within islands of differentiation (Table 1).

**Table 1:** Proportion of amino acid substitutions driven to fixation by positive selection (α) and divergence at nonsynonymous and synonymous sites (dN/dS) for genes within and outside differentiated regions.

Chromosome	Region	α*_O. c. algirus_* b	α*_O. c. cuniculus_* b	dN/dS^c^
Autosomes	Within	0.35	0.85	0.122
		(−0.61;0.96)	(0.26;1.00)	(0.144)
	Outside^a^	0.62	0.67	0.126
		(0.51;0.71)	(0.57;0.72)	(0.114)
X-chromosome	Within	0.97	0.99	0.164
		(0.93;1.00)	(−0.75;1.00)	(0.171)
	Outside^a^	0.97	0.28	0.166
		(0.67;1.00)	(−0.90;1.00)	(0.160)

a Only includes genes located within sampled regions in our study and exclude genes located in regions of differentiation identified using only one of the statistics.

b Method of Eyre-Walker and Keightley [73]. 95% confidence intervals of α estimates were obtained using 200 bootstrap replicates.

c Only genes with one-to-one orthology relationships were considered. Standard deviation is shown in parenthesis.

Single-gene islands provide the strongest candidates for hybrid incompatibilities. Given this, we performed additional examination of their molecular functions (Table 2). We found a wide variety of molecular functions among these candidates, and interactions between distinct elements (e.g. DNA binding, RNA binding, and protein binding), and several transcription activities (e.g. transcription corepressor activity, transcription factor binding, and histone binding), were particularly common. Moreover, several candidate genes were associated with male sterility, which is the only hybrid phenotype detected so far in crosses between rabbit subspecies. For example, EIF4G3 encodes a eukaryotic translation initiation factor, and mutations in this gene have been shown to cause male limited infertility in mice by inducing meiosis and spermatogenesis arrest in meiotic prophase [58]. CDKN2C is a member of the INK4 family of cyclin-dependent kinase inhibitors and its expression is largely confined to spermatocytes undergoing meiosis in seminiferous tubules. Male mice bearing a null CDKN2C mutant suffer from increased germ cell apoptosis, resulting in abnormal spermatogenesis and oligozoospermia [59].

**Table 2:** List of protein coding genes found in regions of differentiation containing a single annotated gene.

Gene	Description	Molecular Function
SPIN1	spindlin 1	DNA binding; methylated histone residue binding
KDM2A	lysine (K)-specific demethylase 2A	DNA binding; protein binding; zinc ion binding; unmethylated CpG binding
ENSOCUG00000021972	NA	zinc ion binding
FAF2	Fas associated factor family member 2	protein binding; ubiquitin protein ligase binding; ubiquitin binding
RB1CC1	RB1-inducible coiled-coil 1	protein binding
XKR4	XK, Kell blood group complex subunit-related family, member 4	NA
DIP2B	DIP2 disco-interacting protein 2 homolog B	transcription factor binding; catalytic activity
SLC4A8	solute carrier family 4, sodium bicarbonate cotransporter, member 8	transporter activity; inorganic anion exchanger activity; anion transmembrane transporter activity
CPSF6	cleavage and polyadenylation specific factor 6	mRNA binding; protein binding
PTPN4	protein tyrosine phosphatase, non-receptor type 4 (megakaryocyte)	protein tyrosine phosphatase activity; receptor activity; cytoskeletal protein binding
PHF14	PHD finger protein 14	zinc ion binding; protein binding
NOTCH2	notch 2	calcium ion binding;protein binding
COL24A1	collagen, type XXIV, alpha 1	extracellular matrix structural constituent
USP24	ubiquitin specific peptidase 24	ubiquitin thiolesterase activity
CDKN2C	cyclin-dependent kinase inhibitor 2C	cyclin-dependent protein kinase inhibitor activity; protein binding
EIF4G3	eukaryotic translation initiation factor 4 gamma, 3	protein binding
TBL1XR1	transducin (beta)-like 1 X-linked receptor 1	DNA binding; transcription corepressor activity; beta-catenin binding; histone binding; transcription regulatory region DNA binding; protein N-terminus binding
CAMK2D	Calcium/calmodulin-dependent protein kinase type II subunit delta	calmodulin-dependent protein kinase activity; calmodulin binding; ATP binding; transferase activity
PCNXL2	pecanex-like 2	NA
TGFB2	Transforming growth factor-beta 2	receptor signaling protein serine/threonine kinase activity; receptor binding; transforming growth factor beta receptor binding; protein binding; growth factor activity; protein N-terminus binding; protein homo and heterodimerization activity
ZNF609	zinc finger protein 609	zinc ion binding
TCF12	transcription factor 12	DNA binding; sequence-specific DNA binding transcription factor activity; protein binding; transcription regulatory region binding; protein heterodimerization activity; E-box binding
CDK12	cyclin-dependent kinase 12	RNA polymerase II carboxy-terminal domain kinase activit; transferase activity, transferring phosphorus-containing groups; cyclin binding; ATP binding; protein binding; protein tyrosine activity
REPS2	RALBP1 associated Eps domain containing 2	calcium ion binding; protein binding
DMD	Dystrophin	zinc ion binding; actin binding; PDZ domain binding; protein binding
IL1RAPL2	interleukin 1 receptor accessory protein-like 2	interleukin-1, Type II, blocking receptor activity; protein binding
AMMECR1	Alport syndrome, mental retardation, midface hypoplasia and elliptocytosis chromosomal region gene 1	NA
POU3F4	POU class 3 homeobox 4	DNA binding (AT and double-stranded); sequence-specific DNA binding transcription factor activity
ENOX2	ecto-NOX disulfide-thiol exchanger 2	nucleic acid binding; protein disulfide oxidoreductase activity
SLC9A6	solute carrier family 9 (sodium/hydrogen exchanger), member 6	sodium:hydrogen antiporter activity

Finally, when we consider our findings in the context of hybrid male sterility genes previously uncovered in laboratory crosses, some intriguing commonalities are apparent. *Prdm9* was the first hybrid male sterility gene discovered in vertebrates and was identified from crosses between mouse subspecies [5]. It contains a KRAB motif, a histone H3 Lysine-4-methyltransferase domain, and several zinc fingers that likely mediate sequence-specific binding to DNA. *OdsH*, a hybrid male sterility gene identified from crosses between *Drosophila mauritiana* and *D. simulans*, is thought to encode a heterochromatin-binding protein, and differential DNA binding properties between alleles result in altered heterochromatic localization and chromosome decondensation [4]. *Ovd*, another hybrid male sterility gene in *Drosophila*, is also thought to function as a DNA-binding protein [6]. It is noteworthy that several genes in our candidate gene list have nucleic-acid binding properties, and at least five genes contain zinc-fingers and two interact with histones similarly to *Prdm9* (Table 2). Crossing experiments and follow-up functional studies will ultimately be necessary to confirm the involvement of these genes in reproductive isolation and to determine the specific phenotypes which they are controlling. The findings presented here provide a manageable list of candidate genes with explicit phenotypic predictions.

## Conclusions

We have used genome-wide data and a population genetic approach to dissect the genetic basis of reproductive isolation in the European rabbit by taking advantage of natural hybridization between subspecies in the Iberian Peninsula. While most of the genome is lowly differentiated between subspecies, we identified numerous regions of strong differentiation, suggesting that the genetic basis of reproductive isolation may be highly polygenic. In addition, the architecture of differentiation is such that these regions were of small size and contained very few genes. This observation and the availability of a reference rabbit genome allowed us to identify several candidate genes for reproductive isolation in this system that suggest an important role for epigenetic and transcription regulation in the maintenance of reproductive barriers. Most interestingly, the molecular functions of some genes identified here using patterns of genetic differentiation in nature strongly parallel those of the hybrid male sterility genes identified using laboratory crosses. Genome-wide analyses of differentiation, such as has been pursued here, are now feasible in a large number of organisms, and are poised to provide new genetic insights into the early stages of speciation.

## Materials and Methods

### Next-generation sequencing

#### Targeted capture

We obtained polymorphism and divergence data from six individuals for each of the rabbit subspecies using Illumina sequencing of genomic regions enriched by DNA hybridization on microarrays [60]. These 12 individuals were selected from 2–3 allopatric populations within the range of each subspecies (Figure S1). A single *Lepus timidus* individual was used as an outgroup. We designed a custom Agilent array for the selective enrichment of 6 Mb of intronic sequence throughout the rabbit genome (5000 fragments of 1.2 Kb; Dataset S2). Given that the current annotation of the rabbit genome is mostly based on computational approaches, we empirically defined intronic DNA from transcriptome sequencing from rabbit brain frontal cortex [28], [29]. Briefly, transcriptome reads were aligned to the rabbit reference genome available in Ensembl (OryCun2.0; www.ensembl.org) using the program Tophat [61] and the exon-exon junctions reported by Tophat used to empirically define intronic regions of the genome. A list of 1.2 kb-wide intronic regions suitable for DNA capture were chosen to be at least 100 bp away from the nearest intron/exon boundary, centered within the given intron, not contain gaps in the genome reference, and to be free of repetitive DNA as defined by both the repeat masker annotation available in the UCSC genome browser (http://genome.ucsc.edu) and a soft-masking strategy [60]. The median physical distance between targets was 268 kb. The preparation of barcoded Illumina sequencing libraries was performed according to Meyer and Kircher [62] and hybridized to the Agilent arrays following the protocol described by Hodges et al. [60]. All sequencing runs were performed on an Illumina GAII platform using a combination of single-end and paired-end 76 bp reads. With the exception of two rabbits and the *Lepus* individual, the remaining samples were sequenced to an effective coverage of at least 30X (i.e. after quality filtering, mapping, and duplicate removal; see below). Further details on capture efficiency are summarized in Table S5. The data were submitted to the Sequence Reads Archive (SRA) of NCBI (accession number: SRP021071).

#### Transcriptome data

To increase the density of SNPs across the rabbit genome, published protein-coding data derived from resequencing the transcriptome of the brain frontal cortex [28], [29] were combined with the data obtained here. Both the transcriptome and the target enrichment datasets were collected using an identical sampling strategy (six individuals from each subspecies collected away from the contact zone) but each dataset derives from a different subset of individuals. Nevertheless, individuals were collected in neighboring localities (Figure S1) and were equivalent in terms of genetic differentiation between subspecies. For example, the average *F_ST_* value among all SNPs (Mean_transcriptome_ = 8.8%, Mean_target enrichment_ = 8.0%) and the ratio of fixed to shared polymorphisms (0.011 vs 0.009) were similar in both datasets. Additionally, the average *F_ST_* values among SNPs contained within 100 kb non-overlapping windows derived independently from each datasets were highly significantly correlated (*R* = 0.5; *P*<1.0×10^−16^; data not shown). Therefore, we have chosen to combine both datasets in all analyses.

#### Base calling, read mapping, and genotype calling

Base calling was performed with the machine learning algorithm Ibis [63] and overlapping read pairs obtained from paired-end sequencing runs were merged into single sequences. Read mapping to the rabbit reference genome (OryCun2.0) was then carried out using the Burrows-Wheeler alignment [64] using default parameters. Prior to SNP calling, PCR duplicates were removed by collapsing molecules with identical mapping coordinates and the read with the highest summed base qualities was kept for further analyses. For the transcriptome dataset, we trimmed the first and last six bases from each aligned molecule to avoid problems that may be caused by a nonrandom composition of the hexamer pools during cDNA synthesis. After both quality control steps, multi-sample SNP/genotype calling was carried out using the algorithm implemented in Samtools [65] using the following criteria. Only SNPs with a minimum quality of 20, RMS minimum mapping quality of 20, and distancing 10 bp from indel polymorphisms were interrogated for individual genotypes. Homozygote and heterozygote genotypes were accepted for each SNP and each individual if the total effective sequence coverage was equal to or higher than 8X and genotype quality equal to or higher than 20, otherwise that specific genotype was coded as missing data. The sequence coverage for the *Lepus* individual was relaxed to 4X. Sequence coverage in regions immediately flanking our hybridization targets was in several cases higher than 8X, therefore, we extended SNP calling to 250 bp of flanking sequence in all targets. SNPs with more than two missing genotypes in either subspecies were removed from the dataset.

The quality thresholds used here produced levels and patterns of polymorphism for the 5,000 intronic fragments qualitatively similar to previous studies using a smaller dataset of intronic fragments and Sanger sequencing [21], [26] (Table S6). Two overall trends were also in agreement with these previous studies. First, *O. c. algirus* is more polymorphic than *O. c. cuniculus*. Second, there is a skew towards rare polymorphisms in both subspecies and this skew is more pronounced in *O. c. algirus*. This skew towards rare variants has been detected even when PCR products form intronic sequences were Sanger sequenced from both strands and from independent PCR reactions [21]. Therefore, the high proportion of rare polymorphisms in our dataset likely reflects the true distribution of allele frequencies and cannot be simply attributed to a higher Illumina sequencing or genotype calling error. Estimates of synonymous site diversity for the transcriptome dataset followed these same overall trends but were consistently higher than estimates for intronic sequences, which is most likely explained by less selective constraint in synonymous sites than in intronic sites as observed for numerous species [e.g. 66]. Alternatively, this difference can also be explained by a higher genotyping error rate in the transcriptome dataset than in the targeted capture data due to variance in coverage among transcripts (i.e. our dataset includes genes with varying transcript abundance levels) and inherent errors in transcription. Although very lowly expressed genes were not included in our analysis because we required at least 8X coverage for SNP calling, both these sources of error are expected to have a greater impact on genes with lower levels of expression. However, when we extracted gene expression levels from the brain transcriptome data following the methodology in Carneiro et al. [29], we found that levels of nucleotide diversity and the proportion of low frequency variants between lowly (bottom 5%) and highly (top 5%) expressed genes in our dataset (Table S7) were not significantly different in most comparisons (with the exception of π in *O. c. cuniculus*) nor did they consistently differ in the expected direction when assuming higher error rates in lowly expressed genes. For example, in *O. c. algirus* π was higher for highly expressed genes when compared to lowly expressed genes. These results suggest that this explanation cannot fully account for the differences we observe.

### Data analysis

#### Levels of variation and tests of neutrality

We estimated the neutral mutation parameter (4*N_e_*μ for autosomal loci and 3*N_e_*μ for X-linked loci), where *N*_e_ is the effective population size and μ is the mutation rate per site per generation, using two estimators: Watterson's θ_w_ [67], the proportion of segregating sites in a sample, and π [68], the average number of pairwise differences per sequence in a sample. The frequency spectrum of polymorphisms was summarized by means of Tajima's *D* [69] and Fay and Wu's *H* [44]. For Fay and Wu's H, alleles were polarized into ancestral or derived states using sequence data from *Lepus*.

#### Genome-wide patterns of differentiation

Levels of genetic differentiation between subspecies were summarized using the fixation index (*F_ST_*) [70] and also in terms of fixed, shared and exclusive polymorphisms [71]. Additionally, we calculated the relative node depth (RND) [41] for the 5,000 intronic fragments. RND was obtained by dividing the average number of pairwise differences between rabbit subspecies (*D_xy_*) [68] by *D_xy_* between rabbits and *Lepus*.

To investigate patterns of differentiation across the genome, we carried out a sliding window analysis using several metrics. This sliding analysis should help overcome problems associated with sampling variance due to the reduced number of rabbits sampled or sequencing errors of individual SNPs. First, we averaged *F_ST_* values for all SNPs within a window of 100 kb, iterated every 10 kb. Because low frequency variants have the potential to bias *F_ST_* estimates and are more likely to represent technical artifacts such as sequencing and PCR errors [30], we excluded all SNPs with a MAF below 10% (i.e. singletons and doubletons in our dataset). The relative level of population differentiation of each window compared to genome-wide background was evaluated by calculating the number of standard deviations departing from the global *F_ST_* averaged over all windows (Z-score) (see [16] for a similar approach). We considered a Z-score equal to three or higher as a signal of elevated differentiation. This cutoff is somewhat arbitrary but this level of differentiation is expected to be much larger than the background levels of differentiation between subspecies observed throughout the genome (see Results and Discussion). Second, we calculated the proportion of fixed differences versus shared polymorphisms for each window and compared it to the genome-wide expectation using a Fisher's exact test, and setting the significance threshold at *P*<0.01. Finally, we investigated the total number of fixed differences per window, which is not dependent on a within-population component of variation.

To demarcate genomic islands of differentiation, we identified all single or consecutive windows with elevated differentiation defined both using the ratio of fixed differences to shared polymorphism and *F_ST_* values (i.e. Z-score>3). Windows separated by less than 50 kb were considered a single island. Varying the size of the window between 50 kb and 250 kb or restricting the analysis to SNPs with higher MAF produced qualitatively unchanged results in terms of number and size of regions of differentiation (data not shown).

#### Coalescent simulations

In order to assess whether the ratio of fixed to shared mutations observed between subspecies was more compatible with selection against introgression than with stochastic variation in sampling or coalescent process we performed coalescent simulations using the algorithm implemented in ms [72]. We used a demographic scenario consisting of two populations of a given effective population size that have diverged in the past and have subsequently exchanged genes. The estimates of current and ancestral population sizes, divergence times, and gene flow inferred in previous studies [21], [26] using an Isolation-with-Migration model [36], [37] were used to generate a dataset equivalent to the targeted capture dataset (4,749 and 251 fragments of 1.2 Kb on the autosomes and X-chromosome, respectively). We note that loci characterized by high levels of differentiation, which are likely to be resisting introgression, were included in our estimates from previous studies; therefore, if anything, the estimates of gene flow used in the simulations are likely to be underestimates, and our results should therefore be conservative. A total of 10,000 simulated datasets were produced and for each replicate the ratio of fixed to shared mutations was recorded and then compared to our empirical dataset.

#### Positive selection inferences

To ask whether genes lying within regions of differentiation were significantly more affected by positive selection than the genome-wide average, we used two approaches. First, we used the transcriptome dataset and estimated the proportion of nonsynonymous mutations driven to fixation by positive selection (α) using the method of Eyre-Walker and Keightley [73] and following the procedure in Carneiro et al. [29]. Second, we investigated rates of evolution in a deeper phylogenetic scale by comparing divergence at nonsynonymous and synonymous sites (dN/dS) between rabbits and mice. Values of dN/dS were obtained from Ensembl Build 62 by means of the Biomart tool [74], and only genes with one-to-one orthology relationships were considered.

#### Gene ontology (GO) enrichment analyses

Statistical analyses of functional overrepresentation of genes that were contained or partially overlapped differentiated regions were performed using DAVID 6.7 [75]. GO groups with less than 5 gene members were excluded from the analysis. Due to the more comprehensive GO term annotation of the mouse genome, we attributed GO terms to rabbit genes based on one-to-one orthology relationships with mouse genes. Only genes located within sampled regions in our study were included, and genes located in regions of differentiation identified using one differentiation statistic were excluded from the background list.

## Supporting Information

Dataset S1 Levels of differentiation between subspecies of the European rabbit using 100 Kb windows iterated every 10 Kb.
(XLSX)

Dataset S2 Chromosomal position of the genomic regions targeted with the hybridization capture on microarrays.
(XLS)

Figure S1 Geographical location of the populations sampled for the transcriptome (red circles) and DNA hybridization on microarrays datasets (black circles). Dark and light grey indicate the range of *O. c. algirus* and *O. c. cuniculus*, respectively. The approximate location of the hybrid zone is indicated (adapted from [24]). Numbers in the figure correspond to populations and the individuals sampled are given in parentheses: 1- Pancas (PAN2); 2- Huelva (HUE52); 3- Sevilla (PED8, PED16, PFR11, and PFR15); 4- Zaragoza (ZRG2, ZRG3, ZRG7, and ZRG13); 5- Lleida (LLE10 and LLE11).
(PDF)

Figure S2 Patterns of allele-frequency change across the rabbit hybrid zone. A- Contrasting patterns of introgression between subspecies of the European rabbit summarized with pie charts of allele frequency data across the rabbit hybrid zone and shape of genealogies for four representative loci (DIAPH2, HPRT1, FMR1, and MSN; data from [21], [26]–[27]. Allele frequency shifts for FMR1 and MSN from *O. c. algirus* to *O. c. cuniculus* are abrupt and few introgressed alleles are found in the other subspecies territory. The genealogy shape at DIAPH2 is similar to MSN and FMR1 but there is a smoother transition in allele frequency, consistent with higher introgression. For HPRT1, there was no obvious pattern of clinal variation, and localities at the extremes of Iberia exhibited no strong differences in allele frequencies. The fact that the SNPs used for each of these loci fall on the branch uniting divergent haplogroups, which is consistent with ancient vicariance and reciprocal monophyly being attained in the past before hybridization took place, suggests that high levels of admixture may have eroded any clinal differentiation in HPRT1. This interpretation is supported by very high estimates of gene flow for this locus using an isolation-with-migration model [25]. This type of genealogy with little or no correspondence with geography is frequently observed in rabbits. B- A summary of clinal patterns at 17 loci without constraint to any particular model of monotonic change (data from [27]). Maximum likelihood monotonic clines are fitted using the PAVA algorithm. The consensus zone center is marked with a black arrow. The relative location of the samples used in this study to the clinal variation across the rabbit hybrid zone is highlighted in blue for both ends of the transect. Note that the loci represented are biased towards loci previously ascertained to show sharp allele frequency differences between both ends of the subspecies distributions, and even for some of these we are able to detect introgressed alleles. Wider clines or even absence of clinal variation (see Figure S2A) are the most common patterns in the rabbit hybrid zone, highlighting that our current sampling is adequate to detect differences in introgression levels among loci.
(PDF)

Figure S3 Autosomes and X-chromosome are significantly enriched for fixed differences relative to simulated neutral expectation. Histogram summarizing the proportion fixed differences to shared mutations for 10,000 simulated datasets mimicking the 4,749 and 251 fragments of 1.2 Kb on the autosomes and X-chromosome, respectively. The red arrows indicate the observed values. The demographic scenario consisted of two populations of a given effective population size that have diverged in the past and have subsequently exchanged genes. Estimates of current and ancestral population sizes (*N_e_*_1_ = 1,600,000; *N_e_*_2_ = 780,000; *N_ancestral_* = 470,000), divergence time (1,800,000 years and assuming a generation time of 1 year), and gene flow (2*Nm*1_autosomal_ = 1.69; 2*Nm*2_autosomal_ = 0.83; 2*Nm*1_X-chromosome_ = 0.38; 2*Nm*2_X-chromosome_ = 0.26) were obtained from previous studies and inferred using an Isolation-with-Migration model [21], [26].
(PDF)

Table S1 List of differentiated regions defined using the proportion of fixed differences to shared polymorphisms.
(PDF)

Table S2 List of differentiated regions defined using a Z-score higher than three.
(PDF)

Table S3 List of genes that overlap the 102 candidate genomic regions resulting from the intersection between the regions identified using the proportion of fixed differences to shared polymorphisms and regions defined using a Z-score higher than three.
(PDF)

Table S4 List of GO terms overrepresented in the set of genes found within the 102 candidate regions.
(PDF)

Table S5 Summary of the number of reads and sequence capture efficiency.
(PDF)

Table S6 Summary of the analyses of polymorphism and frequency spectrum tests of neutrality in both rabbit subspecies for three different datasets.
(PDF)

Table S7 Levels and patterns of nucleotide variation at synonymous sites for highly (top 5%) and lowly (bottom 5%) expressed genes. Significant comparisons are highlighted in red.
(PDF)
