# Supplementary figures and images for: The Genomic Architecture of Population Divergence between Subspecies of the European Rabbit
Source: PLoS Genet. 2014 Aug 28;10(8):e1003519. doi: 10.1371/journal.pgen.1003519 (PMC4148185; doi:10.1371/journal.pgen.1003519)

**FIG. S1**

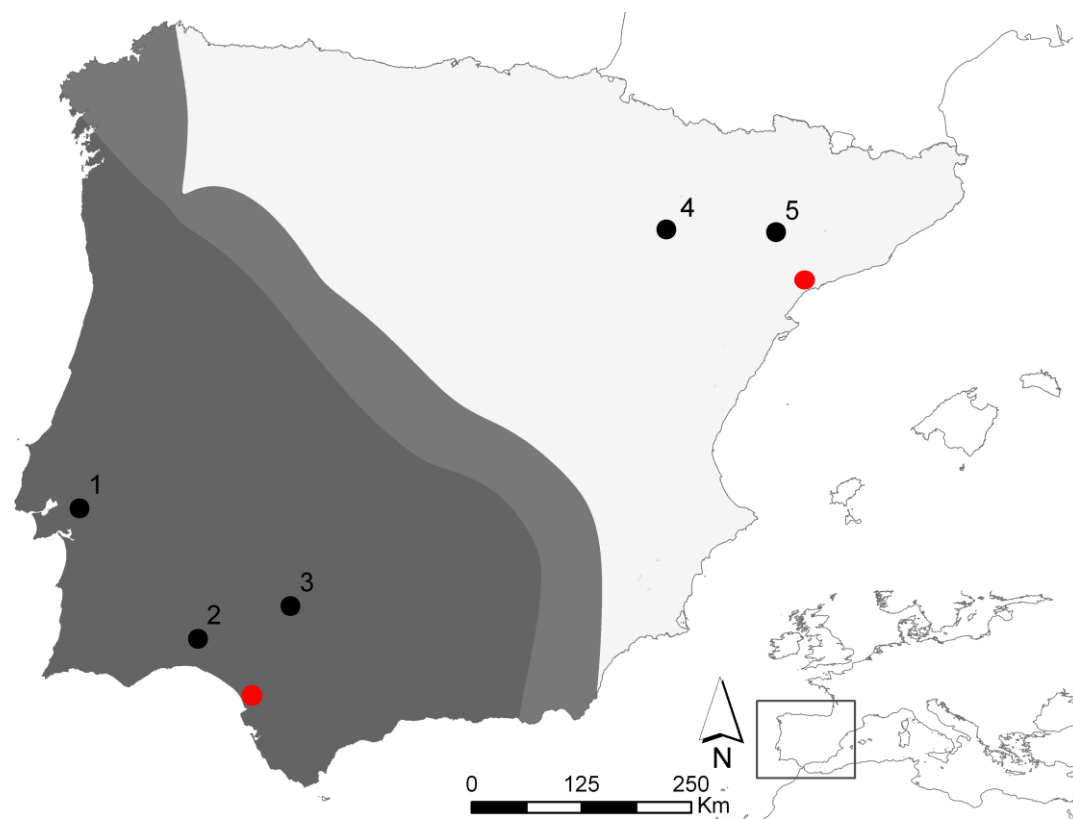

Supplement: Figure S1 — Geographical location of the populations sampled for the transcriptome (red circles) and DNA hybridization on microarrays datasets (black circles). Dark and light grey indicate the range of O. c. algirus and O. c. cuniculus, respectively. The approximate location of the hybrid zone is indicated (adapted from [24]). Numbers in the figure correspond to populations and the individuals sampled are given in parentheses: 1- Pancas (PAN2); 2- Huelva (HUE52); 3- Sevilla (PED8, PED16, PFR11, and PFR15); 4- Zaragoza (ZRG2, ZRG3, ZRG7, and ZRG13); 5- Lleida (LLE10 and LLE11). (PDF) [file pgen.1003519.s003.pdf]

**FIG. S2**

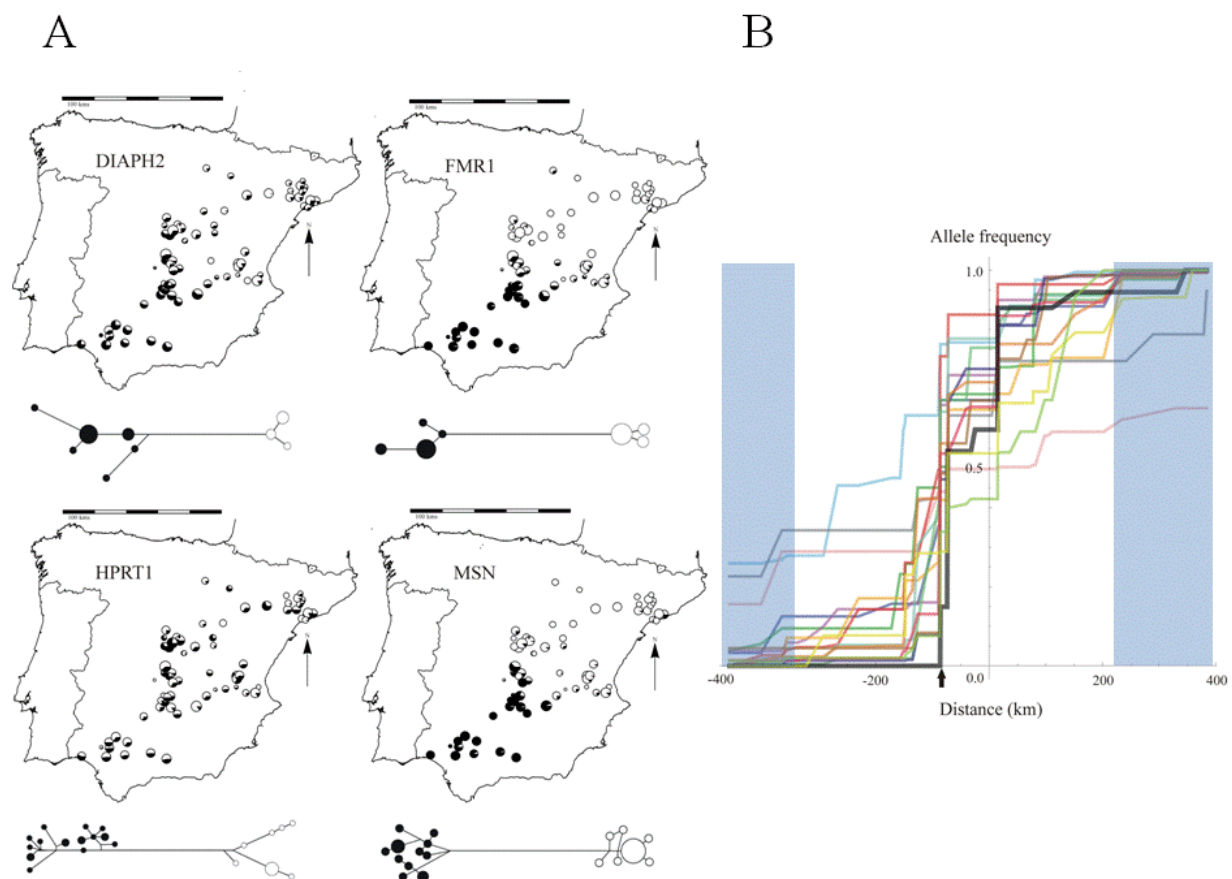

Supplement: Figure S2 — Patterns of allele-frequency change across the rabbit hybrid zone. A- Contrasting patterns of introgression between subspecies of the European rabbit summarized with pie charts of allele frequency data across the rabbit hybrid zone and shape of genealogies for four representative loci (DIAPH2, HPRT1, FMR1, and MSN; data from [21], [26]–[27]. Allele frequency shifts for FMR1 and MSN from O. c. algirus to O. c. cuniculus are abrupt and few introgressed alleles are found in the other subspecies territory. The genealogy shape at DIAPH2 is similar to MSN and FMR1 but there is a smoother transition in allele frequency, consistent with higher introgression. For HPRT1, there was no obvious pattern of clinal variation, and localities at the extremes of Iberia exhibited no strong differences in allele frequencies. The fact that the SNPs used for each of these loci fall on the branch uniting divergent haplogroups, which is consistent with ancient vicariance and reciprocal monophyly being attained in the past before hybridization took place, suggests that high levels of admixture may have eroded any clinal differentiation in HPRT1. This interpretation is supported by very high estimates of gene flow for this locus using an isolation-with-migration model [25]. This type of genealogy with little or no correspondence with geography is frequently observed in rabbits. B- A summary of clinal patterns at 17 loci without constraint to any particular model of monotonic change (data from [27]). Maximum likelihood monotonic clines are fitted using the PAVA algorithm. The consensus zone center is marked with a black arrow. The relative location of the samples used in this study to the clinal variation across the rabbit hybrid zone is highlighted in blue for both ends of the transect. Note that the loci represented are biased towards loci previously ascertained to show sharp allele frequency differences between both ends of the subspecies distributions, and even for some of these we are abl [file pgen.1003519.s004.pdf]

FIG. S3

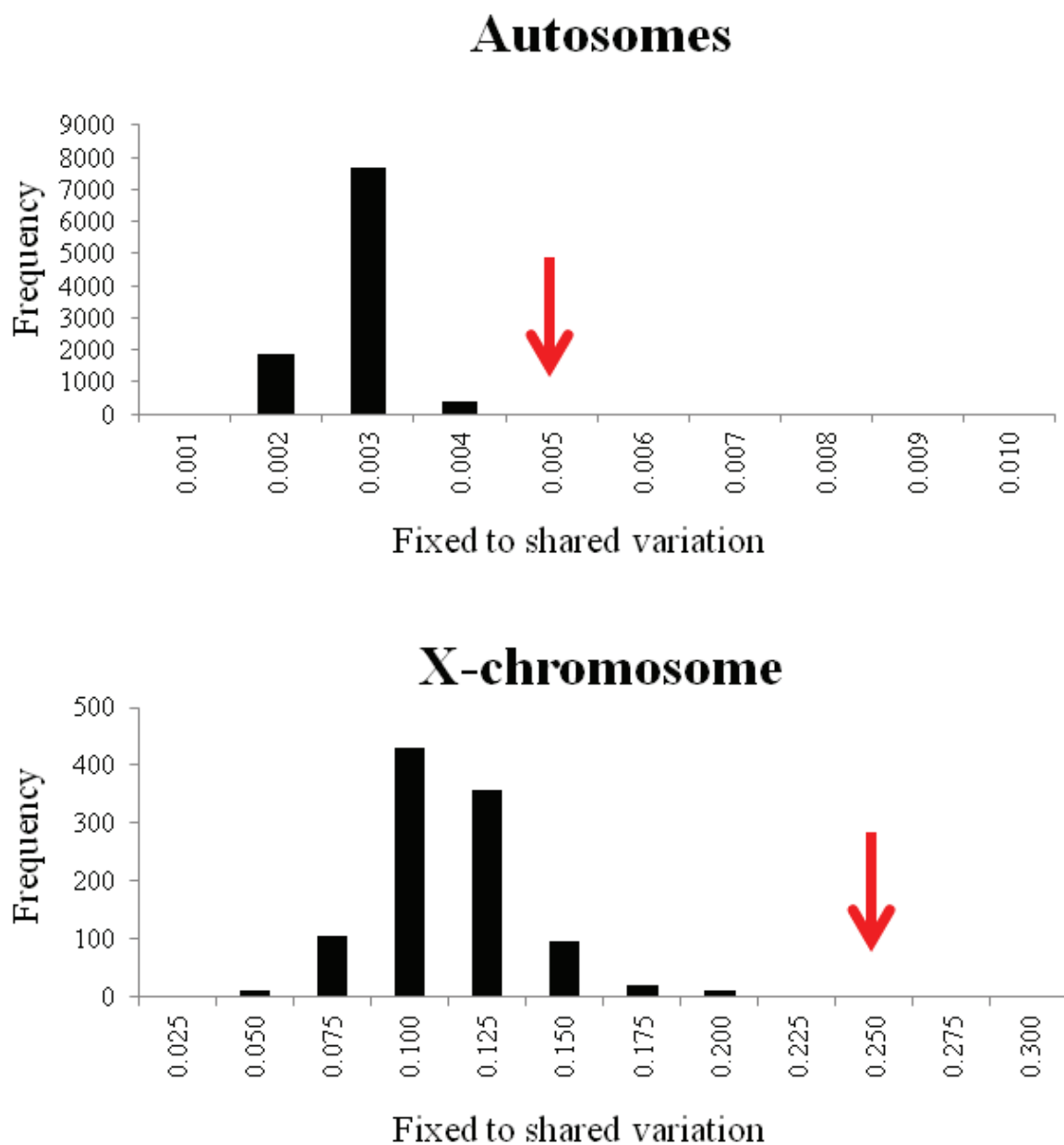

Supplement: Figure S3 — Autosomes and X-chromosome are significantly enriched for fixed differences relative to simulated neutral expectation. Histogram summarizing the proportion fixed differences to shared mutations for 10,000 simulated datasets mimicking the 4,749 and 251 fragments of 1.2 Kb on the autosomes and X-chromosome, respectively. The red arrows indicate the observed values. The demographic scenario consisted of two populations of a given effective population size that have diverged in the past and have subsequently exchanged genes. Estimates of current and ancestral population sizes (Ne 1 = 1,600,000; Ne 2 = 780,000; Nancestral = 470,000), divergence time (1,800,000 years and assuming a generation time of 1 year), and gene flow (2Nm1autosomal = 1.69; 2Nm2autosomal = 0.83; 2Nm1X-chromosome = 0.38; 2Nm2X-chromosome = 0.26) were obtained from previous studies and inferred using an Isolation-with-Migration model [21], [26]. (PDF) [file pgen.1003519.s005.pdf]
