# Supplementary material for: The Genomic Architecture of Population Divergence between Subspecies of the European Rabbit
Source: PLoS Genet. 2014 Aug 28;10(8):e1003519. doi: 10.1371/journal.pgen.1003519 (PMC4148185; doi:10.1371/journal.pgen.1003519)
Supplement: Table S1 — List of differentiated regions defined using the proportion of fixed differences to shared polymorphisms. (PDF) [file pgen.1003519.s006.pdf]

Table S1. List of differentiated regions defined using the proportion of fixed differences to shared polymorphisms.

Chromosome	Region Start (bp)	Region End (bp)	Size of the Window
1	17570000	17760000	190000
1	32940000	33350000	410000
1	74020000	74290000	270000
1	78980000	79170000	190000
1	85580000	85770000	190000
1	114640000	114830000	190000
1	167170000	167360000	190000
1	173460000	173560000	100000
1	194640000	194780000	140000
2	8310000	8500000	190000
2	46290000	46480000	190000
2	66060000	66250000	190000
2	77870000	78060000	190000
2	78410000	78530000	120000
2	115070000	115200000	130000
2	123040000	123230000	190000
2	147250000	147440000	190000
3	14560000	14750000	190000
3	21480000	21670000	190000
3	56930000	57110000	180000
3	57710000	58090000	380000
3	58320000	58730000	410000
3	67490000	67760000	270000
3	70490000	70680000	190000
4	32950000	33780000	830000
4	33850000	34040000	190000
4	34170000	34460000	290000
4	34710000	34900000	190000
4	35370000	36310000	940000
4	36840000	37160000	320000
4	37960000	38090000	130000
4	39450000	39910000	460000
4	43790000	43980000	190000
4	44990000	45180000	190000
4	48100000	48290000	190000
4	81280000	81470000	190000
5	480000	690000	210000
5	27830000	28020000	190000
7	43640000	43780000	140000

7	48860000	49570000	710000
7	62940000	63130000	190000
7	100460000	100650000	190000
7	100950000	101070000	120000
7	110610000	110800000	190000
7	111440000	111540000	100000
8	450000	640000	190000
8	710000	880000	170000
9	18520000	18740000	220000
9	48660000	48810000	150000
9	95740000	95840000	100000
10	27920000	28110000	190000
11	30370000	30560000	190000
12	7220000	7410000	190000
12	16220000	16410000	190000
12	34290000	34480000	190000
12	84430000	85160000	730000
12	107040000	107240000	200000
12	138480000	138670000	190000
13	37010000	37390000	380000
13	37960000	38150000	190000
13	44060000	44250000	190000
13	49370000	49570000	200000
13	63910000	64070000	160000
13	78920000	79110000	190000
13	111690000	111880000	190000
13	111980000	112080000	100000
13	114290000	114490000	200000
13	115430000	115570000	140000
13	115740000	115840000	100000
13	120860000	121060000	200000
13	124260000	124450000	190000
13	132070000	132260000	190000
14	30060000	30500000	440000
14	72300000	72500000	200000
14	118630000	118820000	190000
14	160600000	160790000	190000
15	35720000	35910000	190000
15	76900000	77090000	190000
15	81230000	81460000	230000
16	29600000	29790000	190000
16	30500000	30690000	190000
16	34770000	34960000	190000

16	39380000	39620000	240000
16	54660000	54860000	200000
17	7560000	7750000	190000
17	13460000	13590000	130000
17	15140000	15540000	400000
17	23050000	23240000	190000
17	41360000	41560000	200000
17	71380000	71570000	190000
17	84400000	84510000	110000
18	13900000	14090000	190000
18	22990000	23160000	170000
18	49740000	49940000	200000
18	69100000	69290000	190000
19	15700000	16210000	510000
19	20750000	20920000	170000
19	40730000	40920000	190000
19	41230000	41470000	240000
19	44680000	44860000	180000
19	47580000	47690000	110000
19	50280000	50380000	100000
19	52680000	52870000	190000
20	7810000	8000000	190000
20	10980000	11170000	190000
21	4840000	5060000	220000
21	5490000	5680000	190000
21	7410000	7620000	210000
21	12770000	12960000	190000
X	670000	860000	190000
X	1520000	2110000	590000
X	2830000	3060000	230000
X	4370000	4570000	200000
X	5470000	5660000	190000
X	5940000	7500000	1560000
X	9990000	10540000	550000
X	18790000	18980000	190000
X	33200000	33400000	200000
X	33480000	35250000	1770000
X	35420000	35720000	300000
X	36250000	37510000	1260000
X	41950000	42420000	470000
X	42770000	46560000	3790000
X	47660000	47850000	190000
X	49530000	49920000	390000

X	51860000	52470000	610000
X	52640000	54530000	1890000
X	56350000	56540000	190000
X	58580000	58770000	190000
X	59030000	63140000	4110000
X	68900000	69580000	680000
X	74240000	75320000	1080000
X	86990000	87190000	200000
X	97720000	97990000	270000
X	98310000	98500000	190000
X	99500000	99690000	190000
X	105000000	105190000	190000
X	110480000	110680000	200000
X	110920000	111110000	190000
X	111400000	111550000	150000
