# Supplementary material for: The Genomic Architecture of Population Divergence between Subspecies of the European Rabbit
Source: PLoS Genet. 2014 Aug 28;10(8):e1003519. doi: 10.1371/journal.pgen.1003519 (PMC4148185; doi:10.1371/journal.pgen.1003519)
Supplement: Table S2 — List of differentiated regions defined using a Z-score higher than three. (PDF) [file pgen.1003519.s007.pdf]

Table S2. List of differentiated regions defined using a Z-score higher than three.

Chromosome	Region Start (bp)	Region End (bp)	Size of the Window
1	17650000	17760000	110000
1	66030000	66360000	330000
1	74020000	74290000	270000
1	79050000	79170000	120000
1	92370000	92480000	110000
1	138210000	138400000	190000
1	194620000	194750000	130000
2	8310000	8500000	190000
2	46290000	46480000	190000
2	66150000	66250000	100000
2	77880000	78060000	180000
2	89530000	89720000	190000
2	123040000	125140000	2100000
2	142750000	142940000	190000
2	147250000	147440000	190000
3	14480000	14750000	270000
3	21500000	21610000	110000
3	22590000	22700000	110000
3	53270000	53450000	180000
3	56940000	57040000	100000
3	57710000	58300000	590000
3	58320000	62320000	4000000
3	64420000	64610000	190000
3	67490000	67760000	270000
3	70490000	70680000	190000
3	71220000	71410000	190000
3	74360000	74550000	190000
3	117750000	117850000	100000
3	119410000	119570000	160000
3	123800000	123980000	180000
4	15170000	15360000	190000
4	32950000	33950000	1000000
4	34150000	34460000	310000
4	34710000	34900000	190000
4	35370000	35530000	160000
4	35700000	35890000	190000
4	35980000	37020000	1040000
4	38010000	38120000	110000
4	39450000	39910000	460000

4	40010000	40200000	190000
4	44240000	45180000	940000
4	48110000	48290000	180000
5	27640000	27780000	140000
5	27830000	28020000	190000
7	30920000	31110000	190000
7	47860000	48510000	650000
7	48860000	49370000	510000
7	58700000	58890000	190000
7	59490000	59680000	190000
7	63010000	63130000	120000
7	72150000	72340000	190000
7	90670000	90770000	100000
7	95420000	95550000	130000
7	100460000	100640000	180000
7	111640000	111800000	160000
7	171680000	171880000	200000
8	710000	980000	270000
8	28950000	29050000	100000
8	43860000	44050000	190000
8	44780000	44970000	190000
9	990000	1090000	100000
9	7780000	7940000	160000
9	15650000	15850000	200000
9	17050000	17160000	110000
9	18350000	18740000	390000
9	59430000	59620000	190000
9	65330000	65520000	190000
9	78100000	78290000	190000
9	97940000	98080000	140000
9	110060000	110240000	180000
10	9910000	10030000	120000
10	27920000	28110000	190000
10	30530000	30660000	130000
11	29300000	30560000	1260000
12	24710000	24830000	120000
12	34290000	34480000	190000
12	86450000	86640000	190000
12	111440000	111570000	130000
13	13480000	13770000	290000
13	20300000	21050000	750000
13	22100000	22290000	190000
13	27240000	27340000	100000

13	32820000	32930000	110000
13	37010000	37130000	120000
13	38610000	38720000	110000
13	44060000	44250000	190000
13	49350000	49560000	210000
13	52580000	52680000	100000
13	71990000	72180000	190000
13	78920000	79110000	190000
13	111690000	111880000	190000
13	111980000	112080000	100000
13	114300000	114470000	170000
13	115650000	115830000	180000
13	120860000	121050000	190000
13	122260000	122410000	150000
13	132050000	132250000	200000
13	137330000	137510000	180000
13	138800000	138990000	190000
13	142770000	142960000	190000
14	30060000	30500000	440000
14	46530000	46720000	190000
14	72300000	72500000	200000
14	97460000	97980000	520000
14	98870000	99020000	150000
14	114650000	118820000	4170000
14	121820000	122030000	210000
14	160600000	160790000	190000
15	35720000	36020000	300000
15	39390000	39490000	100000
15	43370000	43560000	190000
15	48410000	48600000	190000
15	81270000	81460000	190000
15	98020000	98250000	230000
16	29600000	29790000	190000
16	30550000	30690000	140000
16	34770000	34960000	190000
16	39380000	39570000	190000
16	54670000	54850000	180000
17	7560000	7930000	370000
17	15140000	15330000	190000
17	23050000	23240000	190000
17	41360000	41560000	200000
17	71380000	71570000	190000
18	13880000	14090000	210000

18	22990000	23180000	190000
18	40390000	40570000	180000
18	49740000	49870000	130000
19	15750000	16130000	380000
19	28140000	28320000	180000
19	29770000	29880000	110000
19	40730000	40850000	120000
19	41300000	41420000	120000
19	41490000	41680000	190000
19	51100000	51290000	190000
19	53760000	53910000	150000
20	7810000	7990000	180000
20	8320000	8420000	100000
20	10990000	11170000	180000
20	31870000	32000000	130000
21	4840000	5030000	190000
21	7460000	7600000	140000
X	2740000	3060000	320000
X	4350000	4570000	220000
X	7000000	7410000	410000
X	10000000	10180000	180000
X	18790000	18980000	190000
X	31790000	31890000	100000
X	33490000	33620000	130000
X	33770000	34290000	520000
X	34550000	34810000	260000
X	34940000	35250000	310000
X	35420000	42140000	6720000
X	42250000	42370000	120000
X	42770000	43680000	910000
X	44460000	44650000	190000
X	47660000	47850000	190000
X	49450000	49810000	360000
X	51860000	52470000	610000
X	52640000	53220000	580000
X	54320000	54530000	210000
X	58590000	58770000	180000
X	59110000	59920000	810000
X	60190000	61510000	1320000
X	64030000	64150000	120000
X	70800000	70990000	190000
X	72500000	72610000	110000
X	74240000	74430000	190000

X	87000000	87190000	190000
X	94640000	97940000	3300000
X	105020000	105190000	170000
X	110490000	110680000	190000
X	110920000	111110000	190000
