# Supplementary material for: The Genomic Architecture of Population Divergence between Subspecies of the European Rabbit
Source: PLoS Genet. 2014 Aug 28;10(8):e1003519. doi: 10.1371/journal.pgen.1003519 (PMC4148185; doi:10.1371/journal.pgen.1003519)
Supplement: Table S3 — List of genes that overlap the 102 candidate genomic regions resulting from the intersection between the regions identified using the proportion of fixed differences to shared polymorphisms and regions defined using a Z-score higher than three. (PDF) [file pgen.1003519.s008.pdf]

Table S3. List of genes that overlap the 102 candidate genomic regions resulting from the intersection between the regions identified using the proportion of fixed differences to shared polymorphisms and regions defined using a Z-score higher than three.

Chromosome	Region Start (bp)	Region End (bp)	Size of the Window	Genes
1	17650000	17760000	110000	ENSOCUG00000000010; ENSOCUG00000014164
1	74020000	74290000	270000	ENSOCUG00000010476; ENSOCUG00000000678; ENSOCUG00000021535; ENSOCUG00000018814; ENSOCUG00000023915
1	79050000	79170000	120000	ENSOCUG00000011880
1	194640000	194750000	110000	ENSOCUG00000009988
2	8310000	8500000	190000	ENSOCUG00000021289; ENSOCUG00000006802; ENSOCUG00000006792
2	46290000	46480000	190000	ENSOCUG00000004114; ENSOCUG00000023197; ENSOCUG00000006355; ENSOCUG00000004102; ENSOCUG00000020861
2	66150000	66250000	100000	No genes annotated
2	77880000	78060000	180000	ENSOCUG00000021972
2	123040000	123230000	190000	ENSOCUG00000018771
2	147250000	147440000	190000	ENSOCUG00000020965; ENSOCUG00000025920; ENSOCUG00000007844; ENSOCUG00000019569
3	14560000	14750000	190000	ENSOCUG00000003842; ENSOCUG00000003847
3	21500000	21610000	110000	ENSOCUG00000026311; ENSOCUG00000006932; ENSOCUG00000019393; ENSOCUG00000028474
3	56940000	57040000	100000	ENSOCUG00000014822
3	57710000	58090000	380000	ENSOCUG00000009182; ENSOCUG00000023209; ENSOCUG00000025618; ENSOCUG00000009622; ENSOCUG00000026152; ENSOCUG00000002783; ENSOCUG00000005920; ENSOCUG00000003496; ENSOCUG00000021584; ENSOCUG00000002779;

				ENSOCUG00000002782; ENSOCUG00000012806; ENSOCUG00000022519; ENSOCUG00000002789; ENSOCUG00000002775
3	58320000	58730000	410000	ENSOCUG00000003217; ENSOCUG00000003228; ENSOCUG00000003211; ENSOCUG00000028398
3	67490000	67760000	270000	ENSOCUG00000016393
3	70490000	70680000	190000	ENSOCUG00000017875
4	32950000	33780000	830000	ENSOCUG00000006188; ENSOCUG00000028032; ENSOCUG00000023914; ENSOCUG00000011586; ENSOCUG00000016881; ENSOCUG00000005894; ENSOCUG00000027812; ENSOCUG00000023285; ENSOCUG00000021984; ENSOCUG00000016805; ENSOCUG00000007390; ENSOCUG00000007394; ENSOCUG00000007401; ENSOCUG00000015834; ENSOCUG00000001414; ENSOCUG00000026025; ENSOCUG00000022501; ENSOCUG00000009580; ENSOCUG00000026066; ENSOCUG00000008404; ENSOCUG00000008409; ENSOCUG00000016821; ENSOCUG00000007379; ENSOCUG00000023146; ENSOCUG00000006202; ENSOCUG00000009574; ENSOCUG00000007383; ENSOCUG00000025362; ENSOCUG00000020035; ENSOCUG00000027893; ENSOCUG00000018038
4	33850000	33950000	100000	ENSOCUG00000001754; ENSOCUG00000001750
4	34170000	34460000	290000	ENSOCUG00000003645; ENSOCUG00000025099; ENSOCUG00000012851; ENSOCUG00000012843; ENSOCUG00000012290; ENSOCUG00000015728; ENSOCUG00000015715; ENSOCUG00000015699; ENSOCUG00000015681; ENSOCUG00000028897
4	34710000	34900000	190000	ENSOCUG00000012013

4	35370000	35530000	160000	ENSOCUG00000009020; ENSOCUG00000009026; ENSOCUG00000013207; ENSOCUG00000021265; ENSOCUG00000019556
4	35700000	35890000	190000	ENSOCUG00000016152
4	35980000	36310000	330000	ENSOCUG00000015761; ENSOCUG00000017934; ENSOCUG00000022530; ENSOCUG00000015089; ENSOCUG00000011746; ENSOCUG00000024797; ENSOCUG00000013920; ENSOCUG00000011070
4	36840000	37020000	180000	ENSOCUG00000027691; ENSOCUG00000013484; ENSOCUG00000026693; ENSOCUG00000016826; ENSOCUG00000001412; ENSOCUG00000001419; ENSOCUG00000017421; ENSOCUG00000004445; ENSOCUG00000001415
4	38010000	38090000	80000	ENSOCUG00000013345; ENSOCUG00000022954; ENSOCUG00000013350
4	39450000	39910000	460000	ENSOCUG00000007227; ENSOCUG00000007224; ENSOCUG00000007216; ENSOCUG00000007206; ENSOCUG00000017375; ENSOCUG00000017371; ENSOCUG00000017352; ENSOCUG00000017349; ENSOCUG00000000997; ENSOCUG00000000998; ENSOCUG00000000999; ENSOCUG00000025969; ENSOCUG00000021918; ENSOCUG00000013895; ENSOCUG00000012558; ENSOCUG00000017322; ENSOCUG00000007212; ENSOCUG00000017368; ENSOCUG00000010069; ENSOCUG00000007200; ENSOCUG00000007204; ENSOCUG00000017342; ENSOCUG00000019059; ENSOCUG00000021504
4	44990000	45180000	190000	ENSOCUG00000005909; ENSOCUG00000014492; ENSOCUG00000004595; ENSOCUG00000014489
4	48110000	48290000	180000	ENSOCUG00000013505

5	27830000	28020000	190000	ENSOCUG00000009954; ENSOCUG00000009966; ENSOCUG00000009957; ENSOCUG00000009972
7	48860000	49370000	510000	ENSOCUG00000010337; ENSOCUG00000007998
7	63010000	63130000	120000	ENSOCUG00000008364
7	100460000	100640000	180000	ENSOCUG00000005884; ENSOCUG00000001418; ENSOCUG00000028798
8	710000	880000	170000	ENSOCUG00000013306; ENSOCUG00000003899; ENSOCUG00000028635
9	18520000	18740000	220000	ENSOCUG00000010328; ENSOCUG00000010322; ENSOCUG00000010317; ENSOCUG00000010286; ENSOCUG00000021627
10	27920000	28110000	190000	ENSOCUG00000004678
11	30370000	30560000	30000	ENSOCUG00000012366; ENSOCUG00000009594
12	34290000	34480000	190000	ENSOCUG00000014574; ENSOCUG00000019366
13	37010000	37130000	120000	ENSOCUG00000010191; ENSOCUG00000004785; ENSOCUG00000010186; ENSOCUG00000003552; ENSOCUG00000026145; ENSOCUG00000027971
13	44060000	44250000	190000	ENSOCUG00000016565
13	49370000	49560000	190000	ENSOCUG00000027988; ENSOCUG00000022455; ENSOCUG00000016424; ENSOCUG00000027966; ENSOCUG00000016438; ENSOCUG00000016457;
13	78920000	79110000	190000	ENSOCUG00000003917
13	111690000	111880000	190000	ENSOCUG00000015591
13	111980000	112080000	100000	ENSOCUG00000004544; ENSOCUG00000005180; ENSOCUG00000005190
13	114300000	114470000	170000	ENSOCUG00000000963; ENSOCUG00000000962; ENSOCUG00000000959; ENSOCUG00000000956
13	115740000	115830000	90000	ENSOCUG00000008385
13	120860000	121050000	190000	ENSOCUG00000011433; ENSOCUG00000016604;

				ENSOCUG00000017939; ENSOCUG00000017938; ENSOCUG00000025231; ENSOCUG00000017363; ENSOCUG00000019668
13	132070000	132250000	180000	ENSOCUG00000002451
14	30060000	30500000	440000	ENSOCUG00000012746; ENSOCUG00000010558; ENSOCUG00000018699; ENSOCUG00000028557; ENSOCUG00000021576
14	72300000	72500000	200000	ENSOCUG00000017078
14	118630000	118820000	190000	ENSOCUG00000000133; ENSOCUG00000011426; ENSOCUG00000000131; ENSOCUG00000021390
14	160600000	160790000	190000	ENSOCUG00000029067; ENSOCUG00000014714
15	35720000	35910000	190000	ENSOCUG00000023650
15	81270000	81460000	190000	ENSOCUG00000014604; ENSOCUG00000015886
16	29600000	29790000	190000	ENSOCUG00000009346; ENSOCUG00000024765
16	30550000	30690000	140000	ENSOCUG00000003998; ENSOCUG00000000171
16	34770000	34960000	190000	ENSOCUG00000014363
16	39380000	39570000	190000	ENSOCUG00000006040; ENSOCUG00000022926
16	54670000	54850000	180000	ENSOCUG00000005279
17	7560000	7750000	190000	ENSOCUG00000002325
17	15140000	15330000	190000	ENSOCUG00000017812
17	23050000	23240000	190000	ENSOCUG00000006461; ENSOCUG00000025020
17	41360000	41560000	200000	ENSOCUG00000025483; ENSOCUG00000007106; ENSOCUG00000010784; ENSOCUG00000015029; ENSOCUG00000026171; ENSOCUG00000009914; ENSOCUG00000009922
17	71380000	71570000	190000	ENSOCUG00000012627; ENSOCUG00000012632; ENSOCUG00000012614; ENSOCUG00000012638
18	13900000	14090000	190000	ENSOCUG00000007151; ENSOCUG00000012532;

18	22990000	23160000	170000	ENSOCUG00000012543; ENSOCUG00000012547; ENSOCUG00000005860; ENSOCUG00000028549 ENSOCUG00000006920; ENSOCUG00000006922
18	49740000	49870000	130000	ENSOCUG00000016819; ENSOCUG00000027864; ENSOCUG00000015386; ENSOCUG00000002768; ENSOCUG00000028599
19	15750000	16130000	380000	ENSOCUG00000016100; ENSOCUG00000016056; ENSOCUG00000001979; ENSOCUG00000013018; ENSOCUG00000024363; ENSOCUG00000014256; ENSOCUG00000016112; ENSOCUG00000019577; ENSOCUG00000019564; ENSOCUG00000019455
19	40730000	40850000	120000	ENSOCUG00000000780
19	41300000	41420000	120000	ENSOCUG00000022057; ENSOCUG00000002954; ENSOCUG00000023409
20	7810000	7990000	180000	ENSOCUG00000027752; ENSOCUG00000009955
20	10990000	11170000	180000	ENSOCUG00000005357; ENSOCUG00000005359; ENSOCUG00000003534
21	4840000	5030000	190000	ENSOCUG00000000818; ENSOCUG00000016484; ENSOCUG00000021096; ENSOCUG00000003855; ENSOCUG00000024191; ENSOCUG00000023566
21	7460000	7600000	140000	ENSOCUG00000002855; ENSOCUG00000002851
X	2830000	3060000	230000	ENSOCUG00000014665
X	4370000	4570000	200000	ENSOCUG00000010775; ENSOCUG00000010801; ENSOCUG00000010796
X	7000000	7410000	410000	ENSOCUG00000014660; ENSOCUG00000014678; ENSOCUG00000028681
X	10000000	10180000	180000	ENSOCUG00000003815; ENSOCUG00000028792
X	18790000	18980000	190000	ENSOCUG00000022509
X	33490000	33620000	130000	ENSOCUG00000011709; ENSOCUG00000027418;

				ENSOCUG00000009021; ENSOCUG00000015788; ENSOCUG00000011703; ENSOCUG00000015757; ENSOCUG00000015767; ENSOCUG00000015777; ENSOCUG00000025357
X	33770000	34290000	520000	ENSOCUG00000023722; ENSOCUG00000001393; ENSOCUG00000017336; ENSOCUG00000027237; ENSOCUG00000005231; ENSOCUG00000010135; ENSOCUG00000021800; ENSOCUG00000000066; ENSOCUG00000027728; ENSOCUG00000019284; ENSOCUG00000027288; ENSOCUG00000019088; ENSOCUG00000019249; ENSOCUG00000018286; ENSOCUG00000019262; ENSOCUG00000019217; ENSOCUG00000023821; ENSOCUG00000025256; ENSOCUG00000019234
X	34550000	34810000	260000	ENSOCUG00000006716; ENSOCUG00000010139; ENSOCUG00000027852; ENSOCUG00000027476
X	34940000	35250000	310000	ENSOCUG00000025084; ENSOCUG00000017937; ENSOCUG00000007354; ENSOCUG00000018373; ENSOCUG00000019200
X	35420000	35720000	300000	ENSOCUG00000000004; ENSOCUG00000007408; ENSOCUG00000027678
X	36250000	37510000	1260000	ENSOCUG00000011207; ENSOCUG00000001716; ENSOCUG00000000570; ENSOCUG00000013603; ENSOCUG00000022810; ENSOCUG00000008672; ENSOCUG00000005068; ENSOCUG00000025886; ENSOCUG00000021859; ENSOCUG00000008666; ENSOCUG00000026728; ENSOCUG00000001711; ENSOCUG00000020128
X	41950000	42140000	190000	No genes annotated
X	42770000	43680000	910000	ENSOCUG00000023259; ENSOCUG00000023698;

X	44460000	44650000	190000	ENSOCUG00000001617; ENSOCUG00000028214; ENSOCUG00000001613; ENSOCUG00000013393 ENSOCUG00000022713; ENSOCUG00000004172; ENSOCUG00000018359; ENSOCUG00000024625
X	47660000	47850000	190000	ENSOCUG00000007244; ENSOCUG00000023534
X	49530000	49810000	280000	ENSOCUG00000012396; ENSOCUG00000027545; ENSOCUG00000004588; ENSOCUG00000014919; ENSOCUG00000013046; ENSOCUG00000025850; ENSOCUG00000006797; ENSOCUG00000006811; ENSOCUG00000016643; ENSOCUG00000021848
X	51860000	52470000	610000	ENSOCUG00000024276; ENSOCUG00000009801; ENSOCUG00000002565; ENSOCUG00000027857; ENSOCUG00000013356; ENSOCUG00000007414; ENSOCUG00000025157
X	52640000	53220000	580000	ENSOCUG00000024906; ENSOCUG00000003541; ENSOCUG00000014228; ENSOCUG00000020863; ENSOCUG00000028958
X	54320000	54530000	210000	ENSOCUG00000002614
X	58590000	58770000	180000	ENSOCUG00000016969
X	59110000	59920000	810000	ENSOCUG00000005540; ENSOCUG00000007042; ENSOCUG00000006145; ENSOCUG00000007033
X	60190000	61510000	1320000	ENSOCUG00000027921; ENSOCUG00000005364; ENSOCUG00000009237; ENSOCUG00000009245; ENSOCUG00000013710; ENSOCUG00000004881; ENSOCUG00000024931; ENSOCUG00000023969; ENSOCUG00000020751; ENSOCUG00000020439; ENSOCUG00000028507
X	74240000	74430000	190000	ENSOCUG00000010927
X	87000000	87190000	190000	ENSOCUG00000010948; ENSOCUG00000013059; ENSOCUG00000002182; ENSOCUG00000005789;

				ENSOCUG00000015256
X	97720000	97940000	220000	ENSOCUG00000000315; ENSOCUG00000027161
X	105020000	105190000	170000	ENSOCUG00000012625
X	110490000	110680000	190000	ENSOCUG00000013167
X	110920000	111110000	190000	ENSOCUG00000022548; ENSOCUG00000012570; ENSOCUG00000028060; ENSOCUG00000006037
