# Supplementary material for: The Genomic Architecture of Population Divergence between Subspecies of the European Rabbit
Source: PLoS Genet. 2014 Aug 28;10(8):e1003519. doi: 10.1371/journal.pgen.1003519 (PMC4148185; doi:10.1371/journal.pgen.1003519)
Supplement: Table S4 — List of GO terms overrepresented in the set of genes found within the 102 candidate regions. (PDF) [file pgen.1003519.s009.pdf]

**Table S4. List of GO terms overrepresented in the set of genes found within the 102 candidate regions.**

| Category | Term description | Term | *P*-value | Fold Enrichment | Count |
|---|---|---|---|---|---|
| Biological process | regulation of transcription | GO:0045449 | 0.00434 | 1.472 | 48 |
| Biological process | transcription | GO:0006350 | 0.00607 | 1.521 | 40 |
| Biological process | chromatin organization | GO:0006325 | 0.01394 | 2.313 | 12 |
| Biological process | nuclear-transcribed mRNA catabolic process, nonsense-mediated decay | GO:0000184 | 0.01706 | 7.083 | 4 |
| Biological process | nuclear-transcribed mRNA catabolic process | GO:0000956 | 0.02733 | 5.964 | 4 |
| Biological process | actin filament-based process | GO:0030029 | 0.02919 | 2.452 | 9 |
| Biological process | chromatin modification | GO:0016568 | 0.02976 | 2.285 | 10 |
| Biological process | macromolecule catabolic process | GO:0009057 | 0.03071 | 1.657 | 20 |
| Biological process | regulation of translation | GO:0006417 | 0.03734 | 3.207 | 6 |
| Biological process | chromosome organization | GO:0051276 | 0.04232 | 1.879 | 13 |
| Biological process | mRNA catabolic process | GO:0006402 | 0.0451 | 4.927 | 4 |
| Molecular Function | ubiquitin thiolesterase activity | GO:0004221 | 0.0019 | 5.198 | 7 |
| Molecular Function | guanyl-nucleotide exchange factor activity | GO:0005085 | 0.00635 | 3.211 | 9 |
| Molecular Function | thiolester hydrolase activity | GO:0016790 | 0.00723 | 4.006 | 7 |
| Molecular Function | DNA binding | GO:0003677 | 0.01943 | 1.412 | 40 |
| Molecular Function | vitamin B6 binding | GO:0070279 | 0.02716 | 4.293 | 5 |
| Molecular Function | pyridoxal phosphate binding | GO:0030170 | 0.02716 | 4.293 | 5 |
| Molecular Function | cofactor binding | GO:0048037 | 0.04543 | 2.113 | 10 |
| Molecular Function | protein domain specific binding | GO:0019904 | 0.04601 | 2.415 | 8 |
| Cellular component | membrane-enclosed lumen | GO:0031974 | 0.00705 | 1.656 | 29 |
| Cellular component | nuclear lumen | GO:0031981 | 0.00904 | 1.769 | 23 |
| Cellular component | nucleoplasm | GO:0005654 | 0.00979 | 2.002 | 17 |

| | | | | | |
|---|---|---|---|---|---|
| Cellular component | organelle lumen | GO:0043233 | 0.01536 | 1.595 | 27 |
| Cellular component | intracellular organelle lumen | GO:0070013 | 0.01536 | 1.595 | 27 |
| Cellular component | nucleoplasm part | GO:0044451 | 0.0328 | 1.884 | 14 |
| Cellular component | plasma membrane part | GO:0044459 | 0.04081 | 1.39 | 33 |
