# Supplementary material for: The Genomic Architecture of Population Divergence between Subspecies of the European Rabbit
Source: PLoS Genet. 2014 Aug 28;10(8):e1003519. doi: 10.1371/journal.pgen.1003519 (PMC4148185; doi:10.1371/journal.pgen.1003519)
Supplement: Table S5 — Summary of the number of reads and sequence capture efficiency. (PDF) [file pgen.1003519.s010.pdf]

Table S5. Summary of the number of reads and sequence capture efficiency.

Sample ID	Species/Subspecies	Number of reads after mapping and duplicate removal	Percentage of reads mapping to targets[a]	Mean read depth among targeted positions	Percentage of targeted positions with at least one mapped read	Percentage of targeted positions with a sequence coverage ≥ 8X
HUE52	*O. c. algirus*	17,506,287	0.17	40	0.998	0.974
PAN2	*O. c. algirus*	16,022,802	0.16	33	0.999	0.963
PED8	*O. c. algirus*	14,159,013	0.18	34	0.998	0.964
PED16	*O. c. algirus*	13,204,738	0.20	34	0.998	0.969
PFR11	*O. c. algirus*	13,263,605	0.17	30	0.998	0.956
PFR15	*O. c. algirus*	16,326,838	0.17	37	0.998	0.972
LLE10	*O. c. cuniculus*	18,203,833	0.18	44	0.999	0.977
LLE11	*O. c. cuniculus*	8,344,822	0.18	20	0.996	0.895
ZRG2	*O. c. cuniculus*	11,125,191	0.17	26	0.998	0.934
ZRG3	*O. c. cuniculus*	18,488,930	0.15	35	0.986	0.859
ZRG7	*O. c. cuniculus*	14,836,863	0.17	35	0.998	0.967
ZRG13	*O. c. cuniculus*	18,062,081	0.17	42	0.999	0.978
LT2012	*Lepus timidus*	7,520,483	0.20	19	0.945	0.832*

[a]After duplicate removal.

*Sequence coverage higher than 4X.
