# Supplementary material for: The Genomic Architecture of Population Divergence between Subspecies of the European Rabbit
Source: PLoS Genet. 2014 Aug 28;10(8):e1003519. doi: 10.1371/journal.pgen.1003519 (PMC4148185; doi:10.1371/journal.pgen.1003519)
Supplement: Table S6 — Summary of the analyses of polymorphism and frequency spectrum tests of neutrality in both rabbit subspecies for three different datasets. (PDF) [file pgen.1003519.s011.pdf]

Table S6. Summary of the analyses of polymorphism and frequency spectrum tests of neutrality in both rabbit subspecies for three different datasets.

Type of data/Site class	Subspecies	Locus	π (%)[a]	θ_w (%)[b]	D_T[c]
Sanger[1]	*O. c. algirus*	Autosomes	0.633	0.777	-0.77
Introns		X-chromosome	0.420	0.471	-0.58
	O. c. cuniculus	Autosomes	0.617	0.677	-0.39
		X-chromosome	0.358	0.381	-0.27
Illumina - Sequence capture[2]	*O. c. algirus*	Autosomes	0.674	0.775	-0.61
Introns		X-chromosome	0.312	0.355	-0.55
	O. c. cuniculus	Autosomes	0.508	0.537	-0.26
		X-chromosome	0.197	0.200	-0.03
Illumina - Transcriptome[3]	*O. c. algirus*	Autosomes	0.829	0.931	-0.38
Synonymous sites		X-chromosome	0.416	0.434	-0.12
	O. c. cuniculus	Autosomes	0.756	0.842	-0.36
		X-chromosome	0.385	0.388	-0.32

[1]Unweighted average values across 27 X-linked loci and 17 autosomal loci. Values obtained from Carneiro *et al.* [26].
[2]Unweighted average values across all genes. Only positions with data for all individuals were considered. by bootstrapping a 1,000 times by locus
[3]Unweighted average values across all fragments. Only positions with data for all individuals were considered. by bootstrapping a 1,000 times by locus
[a]Average number of pairwise differences in a sample [68].
[b]Proportion of segregating sites in a sample [67].
[c]Tajima's *D* [69].
