# Supplementary material for: The Genomic Architecture of Population Divergence between Subspecies of the European Rabbit
Source: PLoS Genet. 2014 Aug 28;10(8):e1003519. doi: 10.1371/journal.pgen.1003519 (PMC4148185; doi:10.1371/journal.pgen.1003519)
Supplement: Table S7 — Levels and patterns of nucleotide variation at synonymous sites for highly (top 5%) and lowly (bottom 5%) expressed genes. Significant comparisons are highlighted in red. (PDF) [file pgen.1003519.s012.pdf]

**Table S7. Levels and patterns of nucleotide variation at synonymous sites for highly (top 5%) and lowly (bottom 5%) expressed genes. Significant comparisons are highlighted in red.**

| Subspecies | Expression category | FPKM[a] | π(%)[b] | $D_t$[c] |
|---|---|---|---|---|
| *O. c. algirus* | Highly | 552.4 | 0.912 | -0.447 |
| | Lowly | 8.0 | 0.863 | -0.318 |
| *O. c.cuniculus* | Highly | 582.7 | 0.626 | -0.445 |
| | Lowly | 7.8 | 0.870 | -0.217 |

Unweighted average values among genes. Genes with less than 50 bp of synonymous sites were excluded.

[a]Median fragments per kilobase of gene model and million reads values among all *O. c. algirus* and *O. c. cuniculus* individuals and averaged across genes for each expression category.

[b]Average number of pairwise differences in a sample [68].

[c]Tajima's *D* [69].
